# Complete Genome Sequence of the Biocontrol Strain *Pseudomonas protegens* Cab57 Discovered in Japan Reveals Strain-Specific Diversity of This Species

**DOI:** 10.1371/journal.pone.0093683

**Published:** 2014-04-02

**Authors:** Kasumi Takeuchi, Naomi Noda, Nobutaka Someya

**Affiliations:** 1 Plant-Microbe Interactions Research Unit, National Institute of Agrobiological Sciences, Tsukuba, Ibaraki, Japan; 2 Hokkaido Agricultural Research Center, National Agriculture and Food Research Organization, Memuro-cho, Kasai-gun, Hokkaido, Japan; Belgian Nuclear Research Centre SCK/CEN, Belgium

## Abstract

The biocontrol strain *Pseudomonas* sp. Cab57 was isolated from the rhizosphere of shepherd’s purse growing in a field in Hokkaido by screening the antibiotic producers. The whole genome sequence of this strain was obtained by paired-end and whole-genome shotgun sequencing, and the gaps between the contigs were closed using gap-spanning PCR products. The *P.* sp. Cab57 genome is organized into a single circular chromosome with 6,827,892 bp, 63.3% G+C content, and 6,186 predicted protein-coding sequences. Based on 16S rRNA gene analysis and whole genome analysis, strain Cab57 was identified as *P. protegens*. As reported in *P. protegens* CHA0 and Pf-5, four gene clusters (*phl, prn, plt*, and *hcn*) encoding the typical antibiotic metabolites and the reported genes associated with Gac/Rsm signal transduction pathway of these strains are fully conserved in the Cab57 genome. Actually strain Cab57 exhibited typical Gac/Rsm activities and antibiotic production, and these activities were enhanced by knocking out the *retS* gene (for a sensor kinase acting as an antagonist of GacS). Two large segments (79 and 115 kb) lacking in the Cab57 genome, as compared with the Pf-5 genome, accounted for the majority of the difference (247 kb) between these genomes. One of these segments was the complete rhizoxin analog biosynthesis gene cluster (ca. 79 kb) and another one was the 115-kb mobile genomic island. A whole genome comparison of those relative strains revealed that each strain has unique gene clusters involved in metabolism such as nitrite/nitrate assimilation, which was identified in the Cab57 genome. These findings suggest that *P. protegens* is a ubiquitous bacterium that controls its biocontrol traits while building up strain-specific genomic repertoires for the biosynthesis of secondary metabolites and niche adaptation.

## Introduction

Many plant commensal strains have been classified into the *Pseudomonas fluorescens* group, which currently includes more than fifty named species [1]. Among the strains in the *P. fluorescens* group, *Pseudomonas protegens* strains Pf-5 and CHA0 (previously called *P. fluorescens* Pf-5 and CHA0, respectively) have been used as model strains in studies on the biosynthesis of several extracellular enzymes such as AprA protease, and secondary metabolites such as 2,4-diacetylphloroglucinol (DAPG), pyrrolnitrin (Prn), and pyoluteorin (Plt), with antibiotic activity in the rhizosphere [2]. These exoproducts contribute to plant protection by these strains and other root-colonizing *Pseudomonas* species with biocontrol activity. *P. protegens* CHA0 was isolated from the roots of tobacco in Swiss soil which was naturally suppressive to black root rot in tobacco caused by *Thielaviopsis basicola* [3]. The fully sequenced strain Pf-5, which is very closely related to strain CHA0, was isolated from the rhizosphere soil surrounding cotton seedlings in Texas, USA [4], [5]. The complete genome data of strain Pf-5 are available at the Pseudomonas.com website (http://www.pseudomonas.com) and provide detailed annotation data of this strain. The genome of strain Pf-5, which is 7.07 Mbp, is the largest *Pseudomonas* genome among *Pseudomonas* spp. (whose genome sizes range from 4.17 Mbp) sequenced to date (http://www.pseudomonas.com). The complete genome data of strain CHA0 have recently been deposited and its size is 6.87 Mbp. Such large genomes should confer an environmental fitness advantage on these strains.

The expression of biocontrol factors depends on the Gac/Rsm signal transduction pathway ([6] and the references cited therein). The Gac/Rsm cascade is initiated by the GacS/GacA two-component system. The GacS sensor kinase is known to be phosphorylated and activates the cognate GacA response regulator by phosphotransfer during slow growth and in the presence of unidentified signal molecules. In *P. protegens* CHA0, activated GacA promotes the transcription of non-coding small RNAs (sRNAs) termed RsmX, RsmY, and RsmZ. These sRNAs have high affinity for the RNA-binding protein RsmA and also for the RsmA paralogue RsmE in strain CHA0 [7]–[10]. The RsmA and RsmE proteins repress the translation of genes involved in secondary metabolism during trophophase. When RsmX/Y/Z sRNAs are induced in idiophase, they relieve the translational repression of target genes by sequestering the RsmA and RsmE proteins, thereby allowing the synthesis of secondary metabolites [9], [11], [12]. Thus, mutants defective in the Gac/Rsm signal transduction pathway have a reduced ability to produce such biocontrol factors and to suppress plant diseases [11], [13]. Two additional sensors termed RetS and LadS were shown to provide input into the Gac/Rsm pathway in *P. fluorescens* and *Pseudomonas aeruginosa* [14], [15]. RetS inhibits and LadS activates the activity of the Gac/Rsm pathway and, in this way, both sensors affect the expression of target genes and biocontrol factors [15].

In aspects of the effective and ecological application of biocontrol agents, screening for plant beneficial pseudomonads has led to advances in biocontrol research. Moreover, the genomic sequence of strain Pf-5 has provided new insights into the molecular basis underlying the pseudomonad-mediated suppression of plant disease. For example, novel natural products including the cyclic lipopeptide orfamide A [16], rhizoxin analogs [17] and the insect toxin FitD [18] have been discovered through genomics-guided approaches. Comparative genome analysis among strains in the *P. fluorescens* group has also contributed to the understanding of other different strains [19]. *P. fluorescens* strain A506 is now commercially available in the USA and is sold as BlightBan A506 [20], which is primarily used to suppress bacterial disease fire blight on pears and apples. The complete genome data of strain A506 revealed distinct features that classified this strain into subgroup 3 of the *P. fluorescens* group, whereas strain Pf-5 was classified into subgroup 1 [19].

Although *P. protegens* has been isolated from North America, Europe, and Africa [21], [22], little has been reported on the characterization of *P. protegens* in Asia. In the present study, we aimed to screen biocontrol pseudomonad strains relative to strains Pf-5 and CHA0 from fields in Japan, with expectations that their genome data should contribute to the study of core and accessory genes of the three strains geographically dispersed. We showed that one of the isolates producing DAPG exhibited similar biocontrol potential against Pythium damping-off and root rot in the cucumber to that of strain CHA0. We named this isolate Cab57, which was fully sequenced and identified as *P. protegens*. Cab57 exhibited typical Gac/Rsm activities such as the expression of Rsm sRNA RsmZ and antibiotic production. We also showed that the genomic comparison and characterization of each strain provides new insights into the identity and diversity of this species.

## Results and Discussion

### Isolation of DAPG-producing Pseudomonads from Rhizosphere Soil in Japan

Approximately 2,800 fluorescent pseudomonads were obtained from plant roots. Based on PCR analysis of this collection to screen the *phlD* ^+^ strain, 48 isolates were selected as candidates of DAPG-producing isolates and their production activities were confirmed by thin layer chromatography (TLC) (data not shown). Of the 48 strains, five were PCR-positive for all of the three other antibiotic biosynthetic genes (for pyrrolnitrin, pyoluteorin, and hydrogen cyanide) that are typically found in *P. protegens*. One of the five strains named Cab57, which was isolated from the rhizosphere of shepherd’s purse [*Capsella bursa-pastoris* (L.) Medik.] growing in a field in Hokkaido, was selected for further analysis.

DAPG production levels by strain Cab57 were determined by HPLC. The concentration of DAPG was 248±69 nmol per optical density at 600 nm (OD_600_) in the strain grown in nutrient broth supplemented with 1% glucose to OD_600_ = 4.0 (one OD_600_ unit corresponds to approximately 1×10^9^ cells/mL). The concentration of DAPG in strain CHA0 under the same growth conditions was 208±62 nmol per OD_600_, which suggested that strain Cab57 is a prominent producer of DAPG, similar to strain CHA0.

### Biocontrol Efficacy of Strain Cab57

To investigate the biocontrol activity of strain Cab57 in a natural habitat, we adopted a cucumber-*Pythium ultimum* pathosystem, which enabled us to evaluate plant protection efficacy by measuring the root and shoot weights. Strain Cab57 was as effective a biocontrol agent as strain CHA0, with 95% confidence (Table 1). Furthermore, this strain had a similar root colonization capacity to that of CHA0, both in the presence and absence of *Pythium* (Table 1).

**Table 1:** The suppression of Pythium damping-off and root rot in the cucumber by *Pseudomonas protegens* CHA0 and Cab57.

Bacterial strain added*	*Pythium* added*	Surviving plants per flask (%)**	Shoot fresh weight per flask (g)**	Root fresh weight per flask (g)**	Colonization by *P. protegens* (log_10_ CFU/g of root) ***
None	–	100 a	0.75 a	0.48 a	ND
CHA0	–	100 a	0.73 a	0.50 a	7.43±0.22
Cab57	–	100 a	0.73 a	0.48 a	7.40±0.18
None	+	6 c	0.18 d	0.10 c	ND
CHA0	+	50 b	0.35 c	0.24 b	8.43±0.07
Cab57	+	73 b	0.43 b	0.30 b	8.29±0.10

*P. protegens strains were added at 10^7^ CFU per g of soil contained within 100-mL flasks (30 g of soil per flask), after planting three 92-h-old, sterile-grown cucumber seedlings per flask. *P. ultimum* was added as a millet-seed inoculum at 2.5 g/kg of soil before planting. Plants were harvested after 7 days.

**Data represent the averages of 10 replicates (flasks containing three cucumber plants) per treatment without *P. ultimum* and 16 replicates per treatment with *P. ultimum*. Means within the same column followed by different letters (a–d) are significantly different (*P*<0.05) according to Fisher’s protected LSD test.

***The rhizosphere-stable plasmid pME6031 containing a tetracycline-resistant determinant [49] was introduced as a selective marker into the bacterial strains to determine their root colonization capacity in soil. ND, not detected.

### The Genome Structure of Cab57 Showed Properties Typical of *P. protegens*

We conducted whole-genome sequencing to obtain information on strain Cab57. The Cab57 genome is organized into a single circular chromosome with 6,827,892 bp (Fig. S1) and 63.3% G+C content. The genome is predicted to encode 6,186 proteins. The general genomic features of strain Cab57 as well as those of strains CHA0 and Pf-5 are listed in Table 2.

**Table 2:** General genomic features of *P. protegens* strains Cab57, CHA0, and Pf-5.

*P. protegens*	Genome size, bp	CDS no.	G+Ccontent, %	rRNA genes no.	tRNA genes no.	Accession no.
Cab57	6,827,892	6,186	63.3	16	68	AP014522
CHA0	6,867,980	6,115	63.4	15	68	CP003190
Pf-5	7,074,893	6,108	63.3	16	71	NC004129

To identify species, the 16S rRNA gene (position 125095.126636) of strain Cab57 was applied to a BLAST search and was found to be 100% identical to that of *P. protegens* CHA0 and Pf-5. Strain Cab57 was also characterized using API 20 NE, which revealed the same profile as that of strain CHA0 with the code 0156557. Strain Cab57 was deposited in the MAFF Genebank, National Institute of Agrobiological Sciences as MAFF212077.

Recently, the JSpecies program has been commonly used to compare two genomes for species definition [23]. The whole-genome sequences of *P. fluorescens* group strains are publicly available. Strains of the *P. fluorescens* group used in the genomic comparison are as follows; *P. protegens* Pf-5 [5], *P. protegens* CHA0 (http://www.pseudomonas.com), *P. chlororaphis* subsp. aureofaciens 30–84 [19], *P. fluorescens* Pf0-1 [24], *P. fluorescens* SBW25 [24], and *P. fluorescens* A506 [19]; covering all the three sub-clades of *P. fluorescens* group [19]. The average nucleotide identity calculated with BLAST algorithm (ANIb) values, which provides a numerical and stable species boundary [23], confirmed the adscription of strain Cab57 to *P. protegens* (Table 3); values higher than 96% were found for strains of the same species. Together with ANIb, tetranucleotide frequency correlation coefficients (TETRA) reinforced the objective boundary for species circumscription when TETRA values >0.99 [23]. As shown in Table 3, both strains of *P. protegens* showed values of >0.999, whereas the other strains showed the values of <0.988. A BLASTp search of the three strains revealed that strain Pf-5 was most abundant in its unique coding sequences (CDSs) and strain Cab57 shared more CDSs with strain CHA0 than with strain Pf-5 (Fig. S2). Of the 234 genes specific to strain CHA0, 64 genes belong to the regions associated with mobile genetic elements as mentioned later in Table 4 and 100 genes encode hypothetical proteins of unknown functions.

**Table 3:** Whole genome comparison of strain Cab57 with those of the *Pseudomonas fluorescens* group.

	Strains within the *Pseudomonas fluorescens* group					
	CHA0	Pf-5	30–84	Pf0-1	SBW25	A506
Average Nucleotide Identity to Cab57 (%)	98.47	98.22	84.29	81.06	80.55	80.32
TETRA value to Cab57	0.99992	0.99984	0.98795	0.9256	0.94548	0.94512

**Table 4:** Mobile genetic elements in the *P. protegens* Cab57 genome.

Type	Gene range	5′ end	3′ end	Size (bp)	%GC	Presence of integrase	Similarity to Pf-5 Prophage
Prophage I	1248 to 1284	1398706	1430358	31653	62.7	No	Hybrid of Prophage 01 and 03
Prophage II	2134 to 2148	2346251	2359004	12754	56.6	Yes	Partly homologous to Prophage 04 and 06
Prophage III	3451 to 3525	3840966	3895710	54745	57.6	Yes	Homologous to Prophage 06
Prophage IV	3558 to 3607	3923029	3966962	43934	58.1	Yes	Homologous to Prophage 03
Transposon 1	1981 to 1998	2176361	2198710	22350	50.3	No	None
Transposon 2	3883 to 3893	4318849	4328514	9666	55.5	Yes	None

Four gene clusters (*hcn, plt, prn,* and *phl*) encoding the typical antibiotic metabolites in *P. protegens* were conserved in the genomic sequence of strain Cab57 (Gene ID 2622–2624, 2829–2845, 3743–3746, and 5898–5905, respectively, in Table S1), which supported the results obtained by screening the five strains that were PCR-positive for all four antibiotic biosynthetic genes described above. In strain CHA0, the production of the major extracellular protease AprA, which functions as a biocontrol factor, was also shown to be controlled by the Gac/Rsm signal transduction pathway [25], [26]. Each of ten genomes of the *P. fluorescens* group, including *P. protegens*, has a conserved cluster for this protease [19]. The *aprA* gene cluster is also conserved in strain Cab57 (Gene ID 3232–3236 in Table S1). The reported genes associated with the Gac/Rsm signal transduction pathway were fully conserved in the Cab57 genome with >99% homology to those of Pf-5 (Table S2).

Other typical gene clusters encoding biocontrol factors were also explored (Table S3). Gene clusters for pyoverdine, whose product has been commonly reported in strain Pf-5 and CHA0, were conserved in the Cab57 genome in four different loci (Gene ID 2934–2935, 4179–4198, 4266–4276, and 4286–4288) as reported in Pf-5 [27]. Gene clusters for Enantio-pyochelin, which has been isolated from strain CHA0 and its gene cluster is fully conserved in Pf-5 [28], and for orfamide A, which has been identified in strain Pf-5 from mining of *Pseudomonas* genomes [16], were also conserved in the Cab57 genome (Gene ID 3642–3651 and 2162–2164, respectively). Phenazine is an antibiotic produced by some *Pseudomonas* strains (excluding *P. protegens* Pf-5 and CHA0). The homology search of the gene cluster over the entire genome suggested that the known pathways for the synthesis of phenazine may not be present in the Cab57 strain.

### Rsm sRNA Expression in *P. protegens* Cab57

**Figure 1:**
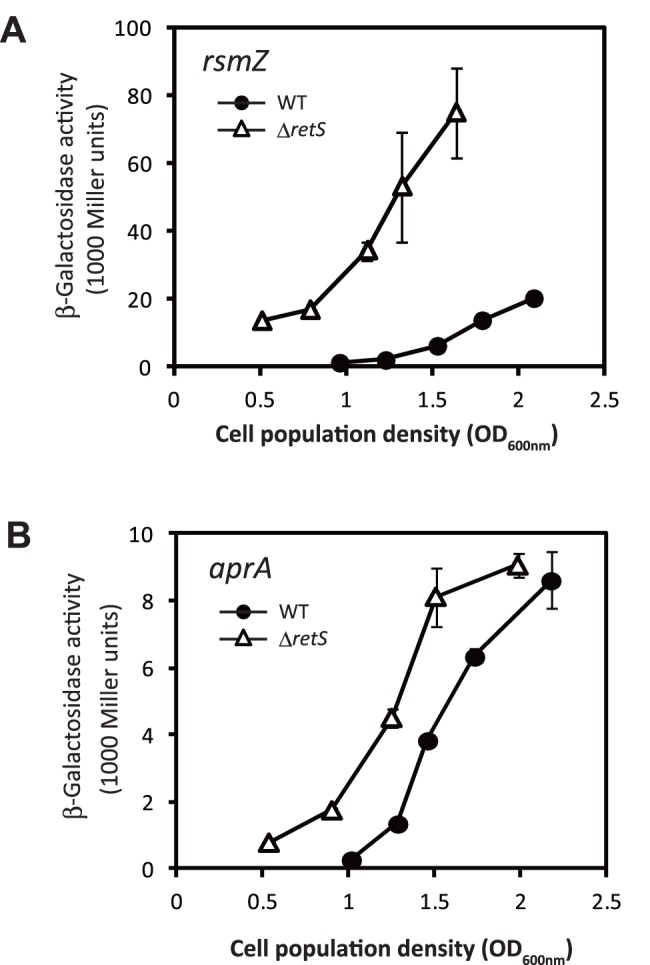
Expression of the *rsmZ*_CHA0_ and *aprA*_ CHA0_ genes in *P. protegens* Cab57. The expression of a *rsmZ*_CHA0_ *-lacZ* fusion carried by pME6091 (A) and *aprA*_CHA0_ *’-‘lacZ* fusion carried by pME6060 (B) was determined in *P. protegens* Cab57 wild type (WT, closed circles) and the *retS* mutant (Δ*retS*, open triangles). The expression of these hybrid proteins was measured according to LacZ activity. Incubation was performed in Erlenmeyer flasks as described in the Materials and Methods. Measurements were conducted in triplicate. Symbols indicate averages and the error bars indicate standard deviations. OD_600 nm_, optical density at 600 nm.

To determine whether *P. protegens* Cab57 conserved the function of the Gac/Rsm cascade, we analyzed the expression of the *rsmZ* sRNA gene. As DNA sequences of the upstream region of *rsmZ* are highly conserved between Cab57 and CHA0 strains (four nucleotides differ in a 200-nucleotide sequence upstream of +1 site, with absolute conservation in the upstream activating sequence (UAS), and in −35, −10, and +1 sites), the transcriptional fusion *rsmZ-lacZ* containing the promoter region of CHA0 was assumed to be applicable to Cab57 by introducing the construct into the strain. As shown in Figure 1, the expression of this fusion developed in a growth-dependent manner, which is consistent with that previously reported for CHA0 [7]. We also speculated as to whether knocking out the *retS* gene (for a sensor kinase acting as an antagonist of GacS) in strain Cab57 would affect the Gac/Rsm cascade. The *retS* mutant showed an enhanced level of expression (Fig. 1), as reported in CHA0 [15], confirming the conserved activity of the Gac/Rsm cascade.

### A *retS* Mutant Exhibited Increased Expression of *aprA* and Antibiotic Activity

The *retS* mutant had been previously reported to cause increased antibiotic production in *P. protegens* CHA0 [15]. In CHA0, the Gac/Rsm system has been shown to positively control the translational expression of *aprA*, the gene for the major exoprotease [29]. As DNA sequences of the upstream region of *aprA* are known to be highly conserved between Cab57 and CHA0 strains (no nucleotide differs in a 200-nucleotide sequence upstream of the start codon), the translational fusion *aprA’-‘lacZ* containing the promoter region of CHA0 was assumed to be applicable to Cab57 by introducing the construct into the strain. Therefore, we followed the expression of *aprA’-‘lacZ* fusion in *P. protegens* Cab57 and the *retS* mutant (Fig. 1). The expression of this fusion again developed in a growth-dependent manner in Cab57 and its expression level was enhanced in the *retS* mutant.

**Figure 2:**
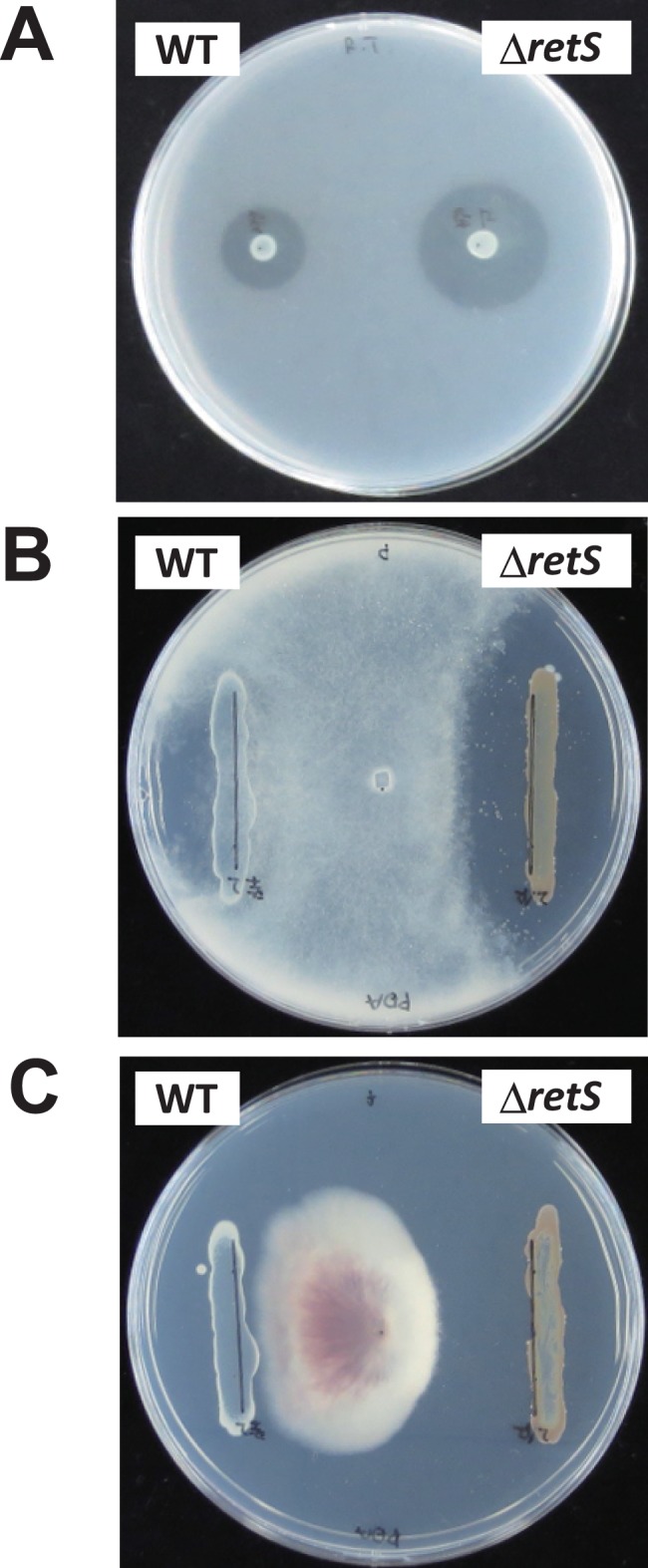
Effects of the *retS* mutation on antibiotic activity. Antibiotic activities of *P. protegens* strains toward *B. subtilis* (A), *P. ultimum* (B) and *F. oxysporum* (C) were evaluated by the size of the growth inhibition zone. Antibiotic activities of *P. protegens* Cab57 wild type (WT) and the *retS* mutant (Δ*retS*) were compared.

We also investigated whether RetS would affect the antibiotic activity of *P. protegens* Cab57. In the *retS* mutant, the production of antibiotics with activity against *Bacillus subtilis* was higher than that of the wild type (Fig. 2A), as reported in CHA0. We also tested the antibiotic activity of this strain and the *retS* mutant toward the phytopathogenic oomycete *Pythium ultimum* on a PDA plate. The growth of *P. ultimum* was inhibited by the wild type Cab57 strain, and was markedly inhibited by the *retS* mutant (Fig. 2B). Growth of the phytopathogenic fungus *Fusarium oxysporum* was also inhibited in a similar manner (Fig. 2C). The pigmentation observed in the *retS* mutant on PDA plates may be attributed to the overproduction of secondary metabolites.

### Whole Genome Comparison

#### Absence of the rhizoxin analogue biosynthesis gene cluster in the Cab57 genome

**Figure 3:**
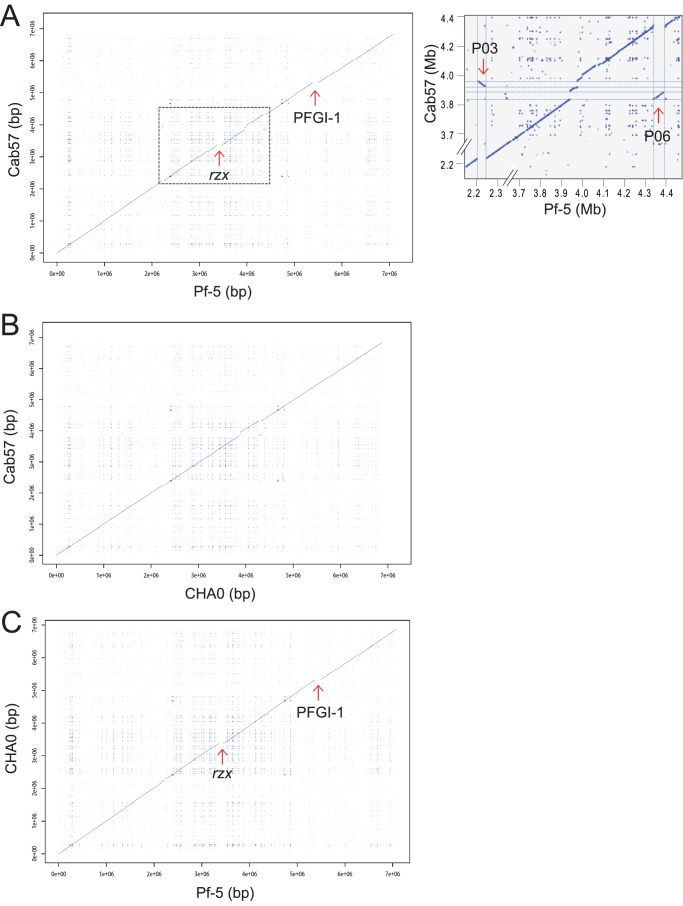
Syntenic dot plot of *P. protegens* strains. Dot plot comparison of the Cab57 genome versus the Pf-5 genome (A), the Cab57 genome versus the CHA0 genome (B) and the CHA0 genome versus the Pf-5 genome (C). The blue line represents the regions of similarities between the two genomes while discontinuities in this syntenic line represent regions of genomic variations at a given locus between the two strains. Positions of phage-related elements and genomic island reported in the Pf-5 genome [34] are marked by arrows (P, Prophage; PFGI, Genomic island). The position of the biosynthetic gene cluster for rhizoxin analogs in the Pf-5 genome [17] is also indicated by an arrow (*rzx*). Regions corresponding to Pf-5 Prophage 03 (P03) and Prophage 06 (P06) in the Cab57 genome are surrounded by a dashed line in panel A and the expanded view is shown in the right.

We performed dotplot analysis to obtain an overview of genome comparisons among the strains of *P. protegens* (Fig. 3). Compared to the Pf-5 genome, whose size is 7,074,893 bp, two large segments were absent in the Cab57 genome (Fig. 3A). These two segments spanning about 195 kb accounted for the majority of the difference (247 kb) between the Cab57 and Pf-5 genomes. These two segments were also absent from the CHA0 genome, the size of which was 6,867,980 (Fig. 3C).

One of these segments was the complete *rzx* gene cluster (ca. 79 kb, Fig. S3), which has been reported to encode genes for the biosynthesis of analogues of the antimitotic macrolide rhizoxin in *P. protegens* Pf-5 [17]. Rhizoxin was firstly isolated from *Rhizopus chinensis*, a fungus causing a disease in rice seedlings, as its virulence factor [30]. Rhizoxin was then found to be produced by *Burkholderia rhizoxinica*, the bacterial endosymbiont of the fungus, but not by *Rhizopus* [31]. The rhizoxin biosynthesis gene cluster (*rhi* gene cluster) was reported in *B. rhizoxinica* [32], and its full genome sequencing has revealed that this cluster is encoded on the chromosome but not in the megaplasmid carried by this strain [33]. However, as the *rhi* gene cluster is flanked by transposase genes, its potential as a mobile region of the genome has been suggested. Furthermore, a trace showed this cluster was originally on one of the megaplasmids of *B. rhizoxinica* [33]. The high conservation of this cluster between two distant species, *P. protegens* Pf-5 and *B. rhizoxinica*, suggests the evolutionary traces of horizontal gene transfer. In the Cab57 genome, the adjacent genes of this cluster (Gene ID 3017 to 3027) were fully conserved with >95% amino acid sequence homologies. Although whether strain Pf-5 acquired this segment, or strain Cab57 discarded this segment after its acquisition remains unclear, such differences in antibiotic production potential indicate diversity among strains.

At the upstream region of this deletion, the *fit* cluster encoding a functional insect toxin reported in *P. protegens* CHA0 [18] was conserved between Cab57 and Pf-5 (Fig. S3, Table S3). This cluster is also conserved in the distant species, *Photorhabdus luminescens*. However, the transmissibility of this cluster has not yet been demonstrated and its evolutionary origin remains unknown.

#### Absence of mobile genomic islands PFGI-1 and -2 of Pf-5 in the Cab57 genome

A 115-kb mobile genomic island 01 of Pf-5 (PFGI-1, [5]) was revealed as another large segment absent from the Cab57 genome (Fig. 3 and Fig. S4). This island was found to be site-specifically integrated into one of the two tRNA genes flanking both sides of PFGI-1 as *attL* and *attR* [5], and its corresponding locus in Cab57 represents the tRNA^Lys^ gene, which is predicted to be a target for the insertion of this island (Fig. S4). PFGI-1 has elements of both temperate phages and conjugative plasmids showing similarity to the mobile genomic island pKLC102 from *P. aeruginosa* C [5], [34]. The *pil* cluster that spans over 10 kb is contained in this island and should encode mating pili rather than type IV pili, and PFGI-1 also carries putative genes involved in conjugal DNA transfer [34]. This *pil* cluster is shared by the pathogenicity islands of *P. aeruginosa* and genomic islands of *P. syringae*. Other notable genes encoded by PFGI-1 such as a cluster of *cyo* genes, which encode components of the cytochrome *o* ubiquinol oxidase complex, have also been predicted to contribute to the survival of *P. protegens* Pf-5; however, their actual function remains unclear [34]. Another genomic island of Pf-5 PFGI-2, which spans 16.8 kb, was also absent from the Cab57 genome. Both of these islands are also absent from the CHA0 genome.

#### Phage-related elements and transposon

**Figure 4:**
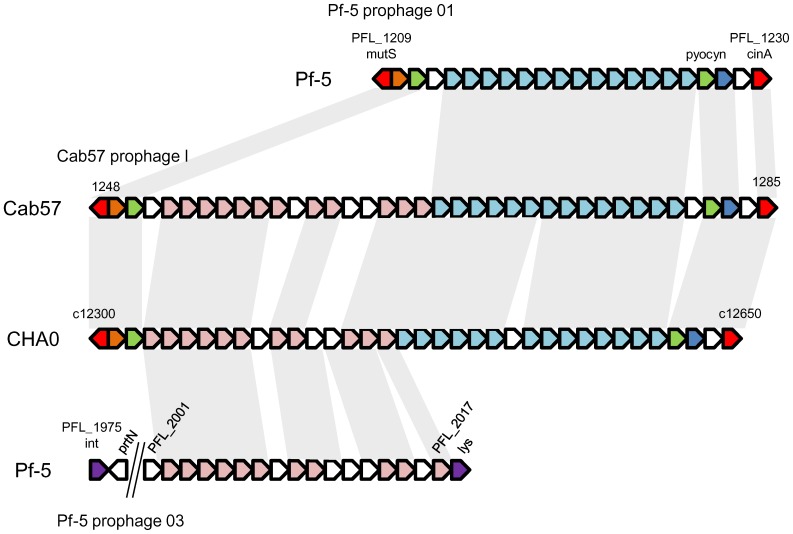
Genetic organization of the region surrounding Prophage I in *P. protegens* Cab57 and the corresponding regions in *P. protegens* CHA0 and Pf-5. The common genes in the three strains are in the same color and conserved genes are connected with gray shading, whereas genes that were not common in all strains are colored white. The sizes of genes are not to scale.

Six prophage regions have been reported as Prophage 01 to 06 in the Pf-5 genome [34]. We identified four prophage regions as Prophage I to IV in the Cab57 genome (Table 4, Fig. 4, Fig. S5, Fig. S6 and Fig. S7). Each prophage showed homology to one or two of the prophages of Pf-5 and was identified occasionally in different integrated sites. For example, Prophage IV, a homologue of Prophage 03 of Pf-5, was identified in a different location of the Cab57 genome where Prophage 05 was identified in the Pf-5 genome (Fig. S7). Two large prophages (Prophage III and IV) were identified in the genomic region of Gene ID 3451 to 3607 (Fig. 3A, Fig. S6 and Fig. S7). The homologue of Prophage 06 was identified in the CHA0 genome in the same integrated sites of the Pf-5 genome (Fig. S6 and Fig. S8), reflecting the continuity in the syntenic line observed by dot plot analysis in the corresponding region (Fig. 3C). The homologue of Prophage 03 was not detected in the CHA0 genome (Fig. S7).

The prophage integrated in the *mutS/cinA* region, which has been reported in ten genomes of the *P. fluorescens* group including Pf-5 [19], was also conserved in the Cab57 genome in the same position at which Prophage 01 was identified in the Pf-5 genome, and was subsequently named Prophage I. Prophage I consists not only of most of the corresponding prophage of Pf-5 Prophage 01 (PFL_1209 to PFL_1230), which closely resembles their counterparts from the long contractile tail of the serotype-converting bacteriophage SfV of *Shigella flexneri* [34], but also the P2-like tail assembly region of Prophage 03 of Pf-5 (PFL_2002 to PFL_2017 of Prophage 03) (Fig. 4). Such a hybrid structure has also been found in a homologous region from *P. fluoresens* Pf0-1, which contains both lambda-like and P2-like tail clusters [34]. The genome of CHA0 also consists of the homologue of Prophage I as a hybrid of Prophage 01 and 03 in the same position as that of Cab57 (Fig. 4).

Prophage II shows homology to the 3′ end of Prophage 04 of Pf-5 and its homologue of CHA0, and three ORFs in Prophage II showed homology to those of Prophage 06 (Fig S5), suggesting the chimeric structure of this prophage. The homologue of Prophage 02 was absent from the Cab57 and CHA0 genomes. The homologue of Prophage 05 was absent from the Cab57 genome, whereas it was present in the CHA0 genome (data not shown).

Unlike the genome of Pf-5, that of Cab57 possesses two IS elements in the genomic regions of Gene ID 1981 to 1998 (Transposon 1) and Gene ID 3883 to 3893 (Transposon 2) with putative transposases (Table S4). Transposon 2 also contains a bacteriophage host lysis protein, suggesting it is a mixture of transposon and prophage. Transposon 2 was identified in the same location at which Prophage 06 of Pf-5 was integrated (Fig. S8). Both of these regions were conserved in the CHA0 genome, whereas several genes including the transposase genes were absent from each region (Table S4).

Overall, the integrated sites of prophages were more restricted in the Cab57 genome than in the Pf-5 genome. These strain-specific segments of the genome were found in limited chromosomal locations, referred to as regions of genomic plasticity.

#### Regions unique to the Cab57 genome

**Figure 5:**
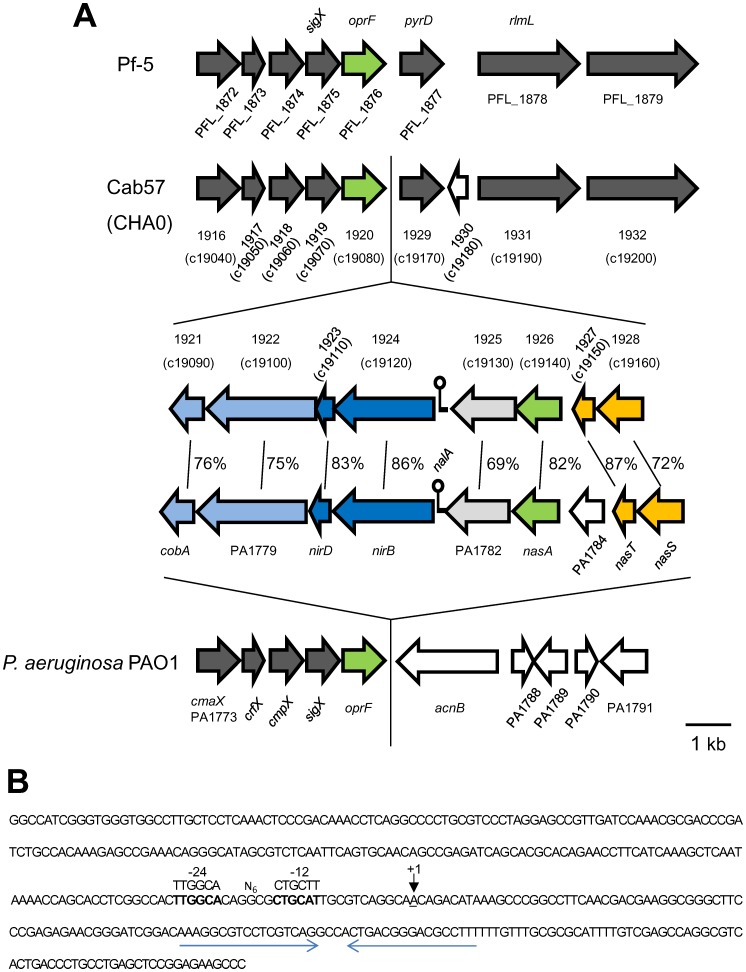
Genetic organization of the nitrite/nitrate assimilation operons in *P. aeruginosa* PAO1 [36], [53] and the corresponding regions in *P. protegens* strains Cab57, CHA0, and Pf-5. (A) ORFs encoding different functional classes of proteins are indicated in each color and the ORFs conserved among the strains are indicated in the same color. The percentage of amino acid identity is indicated. Other conserved genes are colored gray, and strain-specific genes are colored white. (B) Sequence of the intergenic region between Gene ID 1924 and 1925 in *P. protegens* Cab57. The putative −24 and −12 boxes of the *σ* ^54^ -dependent promoter are shown in bold type and the putative transcriptional start site of *nalA* is underlined. The *σ* ^54^ consensus sequence is shown above the sequence. The putative *nalA* terminator sequence is denoted by horizontal arrows.

Apart from the different large genomic islands and prophages found in each genome as described above, we listed regions including at least four contiguous ORFs missing in the Pf-5 genome in order to identify regions unique to Cab57 (as Cluster S1 to S14 in Table S5). One prominent difference in the genome of strain Cab57 from that of Pf-5 was the presence of a putative nitrite/nitrate assimilation operon (Cluster S2: Fig. 5 and Table S5). In *P. aeruginosa* PAO1, the corresponding locus PA1778 to PA1786 was inferred to encode functions required for a nitrite/nitrate assimilation operon, due to homologies with genes reported in other bacteria, and its function has already been demonstrated [35], [36]. For example, the deletion mutant of PA1785, the product of which is required for transcription of the nitrate/nitrite reductase operon, failed to grow on nitrate as the sole nitrogen source [36]. In that study, they found a small RNA termed nitrogen assimilation leader A (NalA) in the intergenic region of PAO1, which is required for the expression of this operon. The NalA homologue was identified in the same locus in the Cab57 genome. The −24 and −12 boxes of the *σ* ^54^ -dependent promoter, both of which are indispensable for promoter activity upon nitrogen depletion, were conserved in its promoter sequence. Unlike PAO1 and *Salmonella typhimurium*, putative palindromic NtrC binding motifs, which are involved in the full expression of the promoter activity necessary [36], [37], were not found. The *nalA* terminator sequence was conserved as a palindromic sequence (Fig. 5). This operon was also present in the CHA0 genome.

*P. protegens* Pf-5 has been reported to be defective in reducing nitrates, which was attributed to the absence of this operon [19]. Only three of the ten sequenced strains in the *P. fluorescens* group (strains O6, Q8r1-96, and Q2-87) possess this operon [19]. The presence of this operon in strain Cab57 suggests that this is not a core component in the same species; therefore, it may provide diversity at the species level.

**Figure 6:**
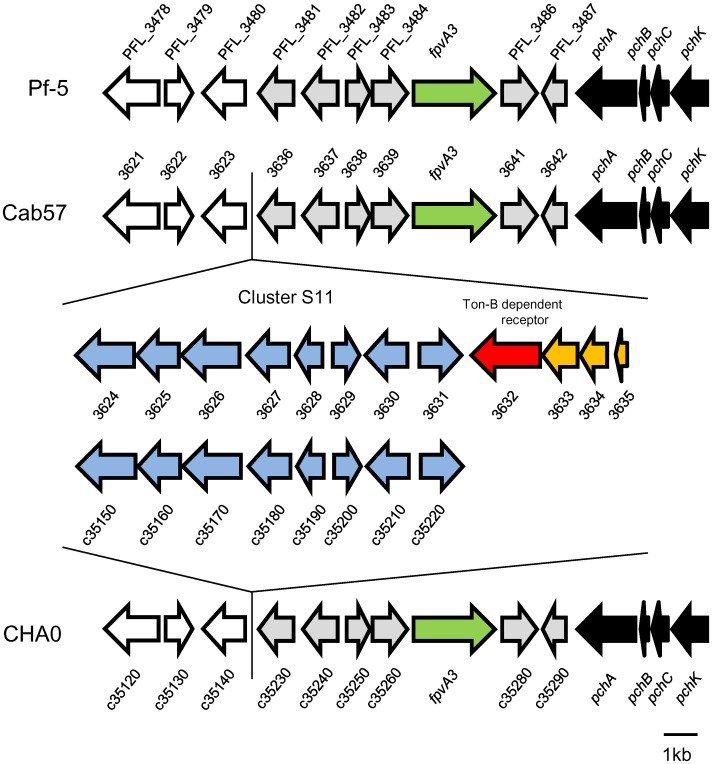
Genetic organization of Cluster S11 in *P. protegens* Cab57 and the corresponding regions in *P. protegens* strains Pf-5 and CHA0. Cluster S11 ORFs conserved in Cab57 and CHA0 genomes are indicated in blue, whereas those specific to the Cab57 genome are indicated in red and yellow. ORFs for the *pch* cluster are in black. The ORF for the ferripyoverdine receptor (fpvA3) is in green, and that for the TonB siderophore receptor is in red. The ORFs conserved among the strains are indicated in the same color. This figure was based on sequence data from the Pseudomonas.com website (http://www.pseudomonas.com) and from Youard et al [28].

Other gene clusters include the following; Cluster S3 encoding the penicillin binding protein, Cluster S10 encoding formate dehydrogenase, Cluster S13 encoding the McrBC restriction enzyme, and Cluster S14 involved in the O-antigen (Table S5). Cluster S11 and Cluster S12 were identified as secondary metabolite clusters by the antiSMASH program. From an ecological viewpoint, such gene clusters may also contribute to niche adaptation by this strain. Cluster S11 (Gene ID 3624 to 3635) was located near the *pch* cluster (Gene ID 3642 to 3651, for enantio-pyochelin), and we identified the PFL_3485 homologue (Gene ID 3640) which was annotated as the ferripyoverdine receptor, between these two clusters (Fig. 6). Cluster S11 possessed a specific region (Gene ID 3632 to 3635) that was also absent from strain CHA0. One of the genes (Gene ID 3632) was annotated as the TonB-dependent siderophore receptor, which suggests different potentials for iron acquisition among the strains.

## Materials and Methods

### Ethics Statement

For the isolation of fluorescent pseudomonads, plant roots were sampled on private lands in diverse locations (Hokkaido, Ibaraki, Kanagawa, Nagano, Aichi and Nagasaki) in Japan after the land owners gave permission to conduct sampling on the sites. None of these species is an endangered or protected species.

### Isolation of DAPG-producing Pseudomonads from Rhizosphere Soil and Selection for Whole Genome Analysis

We isolated approximately 2,800 fluorescent pseudomonads based on a method described previously [38]. DAPG-producing isolates were pre-screened by PCR using the DAPG biosynthesis gene *phlD*-specific primers Phl2a and Phl2b (Table S6). *phlD* ^+^ isolates were incubated on dNBYG medium [39] and DAPG production was evaluated by TLC [40]. DAPG production was also evaluated in strain Cab57 by HPLC [41]. Other antibiotic biosynthetic genes were also explored by PCR analysis using specific sets of primers; PRND1/PRND2 for pyrrolnitrin, PltBf/PltBr for pyoluteorin, and PM2/PM7-26R for hydrogen cyanide (Table S6). Selection by this method revealed five isolates were PCR-positive for all four antibiotic biosynthetic genes and one of the five strains named Cab57, which was isolated from the rhizosphere of shepherd’s purse [*Capsella bursa-pastoris* (L.) Medik.] growing in a field in Hokkaido, was selected for whole genome analysis.

### Bacterial Strains and Growth Conditions

The bacterial strains and plasmids used are listed in Table 5. *E. coli* and *P. protegens* strains were routinely grown in NYB (2.5% [w/v] nutrient broth, 0.5% [w/v] yeast extract) and Luria-Bertani (LB) medium with shaking, or on nutrient agar plates (4% [w/v] blood agar base, 0.5% [w/v] yeast extract) amended with the following antibiotics when required: ampicillin, 100 μg/mL (only for *E. coli*); kanamycin, 25 μg/mL; or tetracycline, 25 μg/mL (125 μg/mL for selection of *P. fluorescens*). The inoculation temperatures were 28°C for *P. protegens* and 37°C for *E. coli. P. protegens* was grown at 33°C to improve its capacity to accept heterologous DNA (e.g., in electrotransformation or in matings with *E. coli*). Bacteria were grown in glycerol-casamino acid medium (GCM) for other assays [42].

**Table 5:** Bacterial strains and plasmids used in this study.

Strain or plasmid	Description	Source or reference
Strains		
* Bacillus subtilis*		
M168	Wild type	C. Keel
* Escherichia coli*		
DH5α, HB101	Laboratory strains	[50]
* Pseudomonas protegens*		
CHA0	Wild type	[3]
Cab57	Wild type	This study
Cab57retS	Δ*retS*	This study
Plasmids		
pCR-Blunt II-TOPO	Cloning vector, pUC *ori*; Km^r^	Invitrogen
pME497	Mobilizing plasmid, IncP-1, Tra, RepA(Ts); Ap^r^	[51]
pME3087	Suicide vector, ColE1 replicon, Mob; Tc^r^	[52]
pME3087retS	pME3087 containing a BamHI/HindIII 1.3-kb *retS* region containing a deletion of the *retS* gene; Tc^r^	This study
pME6031	pACYC177-pVS1 shuttle vector; Tc^r^	[49]
pME6060	Translational *aprA’-‘lacZ* fusion; Tc^r^	[25]
pME6091	Transcriptional *rsmZ-lacZ* fusion; Tc^r^	[7]

### DNA Manipulation

Small-scale plasmid extraction was performed with a QIAprep spin miniprep kit (QIAGEN); large-scale preparations were obtained with a QIAGEN plasmid midi kit (QIAGEN). Chromosomal DNA from *P. protegens* was prepared with a QIAGEN Genomic DNA buffer set and Genomic-tip (QIAGEN). DNA fragments were purified from agarose gels with a QIAquick gel extraction kit (QIAGEN). Oligonucleotides used are listed in Table S6.

### Whole Genome Sequencing and Assembly

Whole genome sequencing was performed using Roche Genome Sequencer FLX Titanium (for 8 kb long paired-end sequencing) and FLX+ (for shotgun sequencing) technology provided by Operon Biotechnologies K.K. (Tokyo, Japan). A shotgun library was prepared and subsequently sequenced, generating 361,203 reads in 211.0 Mbp of sequencing data. An 8 kb long paired-end library was also prepared and sequenced, generating 394,536 reads in 78.5 Mbp of sequencing data. Co-assembly of the results of both shotgun and paired-end sequencing was performed by newbler 2.6, and were fully assembled into 14 large contigs defined as >1 kb. These contigs were then integrated only one scaffold, the size of which was 6.8 Mbp with several gaps. Gaps between the contigs were filled in by sequencing the PCR products using Applied Biosystems 3100×l DNA Analyzer. High-fidelity DNA polymerase KOD Plus™ (Toyobo) was used for PCR amplification. The prediction of putative coding sequences and gene annotation was performed using the Microbial Genome Annotation Pipeline (http://migap.lifesciencedb.jp/). The circular map that illustrates the general genomic feature as well as its comparison with other two strains was plotted by using the software BRIG [43].

### Accession Number

The complete genome sequence of *P. protegens* Cab57 has been deposited in the DDBJ/EMBL/GenBank database under accession no. AP014522.

### Species Identification

To identify species, the 16S rRNA gene of strain Cab57 was applied to a BLAST search. The strain was also characterized using API 20 NE strips (BioMerieux Vitek, Inc.) incubated at 28°C for 48 hours. Whole genome comparisons of strain Cab57 with those of the *P. fluorescens* group were performed using the JSpecies program [23]. This program is commonly used to compare two genomes and uses BLAST software [44].

### Comparative Genome Analysis

To compare the whole genome of *P. protegens* Cab57 with the *P. protegens* Pf-5 or CHA0 strain, the alignment data between each two strains’ genome sequences were prepared using the LASTZ program (Release 1.02.00), and dot plots were then illustrated from the alignment data by R (version 3.0.1). Genomic regions that were unique to each strain were searched for using the Island Viewer program [45] and verified manually. Secondary metabolite production clusters were examined using the antiSMASH program [46].

### Plant Disease Suppression and Root Colonization Assays

Flasks containing vermiculite were planted with three cucumber seedlings each and treated with *Pythium ultimum* MAFF425494 and/or *P. protegens*. After seven days of incubation, the biocontrol activity and root colonization of each strain were estimated as described previously [47].

### Generation of *retS*-negative Mutant

Primers are listed in Supplementary Table S6. An in-frame deletion in the chromosomal *retS* gene of *P. protegens* Cab57 was created as follows. Fragments of 620-bp located upstream and 640-bp located downstream of the *retS* gene, respectively, were amplified by PCR with the primer pairs RetSUF/RetSUR and RetSDF/RetSDR, high-fidelity DNA polymerase KOD Plus™ (Toyobo), and genomic DNA of *P. protegens* Cab57 as a template. Each two corresponding fragments were annealed and amplified as a 1.3-kb fragment using the primer pair RetSUF/RetSDR. This 1.3-kb fragment was cloned into pCR-Blunt II-TOPO™ (Invitrogen). The insert obtained was confirmed by sequencing and digested with BamHI and HindIII. After sequencing, these fragments were subcloned into pME3087 cleaved at the BamHI and HindIII sites to give pME3087retS. This plasmid was mobilized from *E. coli* DH5α to *P. protegens* Cab57 by triparental mating with *E. coli* HB101/pME497. Excision of the vector via a second crossing-over was obtained after the enrichment of tetracycline-sensitive cells, generating the *retS* mutant.

### β-Galactosidase Assays

β-Galactosidase activities were quantified by the Miller method [48]. *P. protegens* strains were grown at 30°C in 50-mL flasks containing 20 mL of NYB amended with 0.05% Triton X-100 with shaking at 180 rpm. Triton X-100 was required to prevent cell aggregation.

### Detection of Antibiotic Activity

The antibiotic activities of *P. protegens* strains were determined with *B. subtilis* M168, *Pythium ultimum* MAFF425494, or *Fusarium oxysporum* MAFF105034 as the reporter. Cultures of *P. protegens* were adjusted to OD_600 nm_ = 1.5 for the assay with *B. subtilis,* and 5-μL samples were spotted onto the GCM plate. After overnight incubation at 30°C, cells were killed by UV irradiation on a transilluminator for 5 min. An overlay of *B. subtilis* revealed antibiotic production by growth inhibition zones. Cultures of *P. protegens* were adjusted to OD_600 nm_ = 1.5 for the assay with *P. ultimum* or *F. oxysporum*, 20 μL samples were streaked around the edge of the potato dextrose agar (PDA) plate, and an inoculum of *P. ultimum* or *F. oxysporum* was transferred to the center of the plate. The plate was incubated at room temperature until *P. ultimum* or *F. oxysporum* reached the edge of the plate.

## Supporting Information

Figure S1 **Circular genome map of** ***P. protegens*** **Cab57.** From the inside out, circle 1 represents the mean centered G+C content (bars facing outside-above mean, bars facing inside-below mean); circle 2 shows the GC skew (G–C)/(G+C); 3 (blue) and 4 (red) represent comparison of gene content with *P. protegens* strain CHA0 and Pf-5, respectively. The outermost ring highlights the regions of mobile genetic elements (P, Prophage shown in green; T, Transposon shown in purple), and gene clusters which are absent from Pf-5 genome (S, shown in black) as described in Table 4 and, respectively. The inner black circle shows the scale line in Mbps.
(EPS)

Figure S2 **Venn diagram comparing the coding sequence sets of** ***P. protegens*** **strains Cab57, CHA0, and Pf-5.** The number of orthologous coding sequences (CDSs) shared by all strains is in the center. The numbers in the non-overlapping portions of each circle represent the number of CDSs unique to each strain. The total number of CDSs within each genome is listed below the strain name. Each set of genes was compared to the other two sets using BLASTp, and sequence identity cut-off was set at 60% to identify common genes. Note that due to multiple hits, gene numbers did not add up to the strain totals.
(TIF)

Figure S3 **Genetic organization of** ***fit*** **cluster (for FitD toxin) and** ***rzx*** **cluster (for rhizoxin analogs) in** ***P. protegens*** **Pf-5 and the corresponding regions in** ***P. protegens*** **Cab57 and CHA0.** The conserved genes are colored gray, and strain-specific genes are colored white.
(TIF)

Figure S4 **Genetic organization of the region surrounding the genomic island PFGI-1 in** ***P. protegens*** **Pf-5 and the corresponding regions in** ***P. protegens*** **Cab57 and CHA0.** The conserved genes are colored gray, and strain-specific genes are colored white.
(TIF)

Figure S5 **Genetic organization of the region surrounding Prophage II in** ***P. protegens*** **Cab57 and the corresponding regions in** ***P. protegens*** **Pf-5 and CHA0.** Homologous genes are connected with gray shading. The genes in prophage are colored white and genes outside prophage are colored gray. The sizes of genes are not to scale.
(TIF)

Figure S6 **Genetic organization of the region surrounding Prophage III in** ***P. protegens*** **Cab57 and the corresponding regions in** ***P. protegens*** **Pf-5 and CHA0.** Homologous genes are connected with gray shading. The genes in prophage are colored white and genes outside prophage are colored gray. The sizes of genes are not to scale.
(TIF)

Figure S7 **Genetic organization of the region surrounding Prophage IV in** ***P. protegens*** **Cab57 and the corresponding regions in** ***P. protegens*** **Pf-5 and CHA0.** Homologous genes are connected with gray shading. The genes in prophage are colored white and genes outside prophage are colored gray. The sizes of genes are not to scale.
(TIF)

Figure S8 **Genetic organization of the region surrounding Transposon 2 in** ***P. protegens*** **Cab57 and the corresponding regions in** ***P. protegens*** **Pf-5 and CHA0.** The conserved genes are colored gray, and strain-specific genes are colored white.
(TIF)

Table S1 **Sequence analysis of gene clusters for the synthesis of antibiotics and exoenzyme in** ***P. protegens*** **Cab57 and similarities to those in** ***P. protegens*** **Pf-5.**
(DOCX)

Table S2 **Sequence analysis of Gac/Rsm homologues in** ***P. protegens*** **Cab57 and similarities to those in** ***P. protegens*** **Pf-5.**
(DOCX)

Table S3 **Sequence analysis of gene clusters for the synthesis of cyclic lipopeptide, siderophores, and toxin in** ***P. protegens*** **Cab57 and similarities to those in** ***P. protegens*** **Pf-5.**
(DOCX)

Table S4 **Sequence analysis of the transposon of the** ***P. protegens*** **Cab57 genome.**
(DOCX)

Table S5 **Sequence analysis of gene clusters in** ***P. protegens*** **Cab57 which are absent from Pf-5 genome.**
(DOCX)

Table S6 **Oligonucleotides used in this study.**
(DOCX)
