# Supplementary figures and images for: Complete Genome Sequence of the Biocontrol Strain Pseudomonas protegens Cab57 Discovered in Japan Reveals Strain-Specific Diversity of This Species
Source: PLoS One. 2014 Apr 2;9(4):e93683. doi: 10.1371/journal.pone.0093683 (PMC3973561; doi:10.1371/journal.pone.0093683)

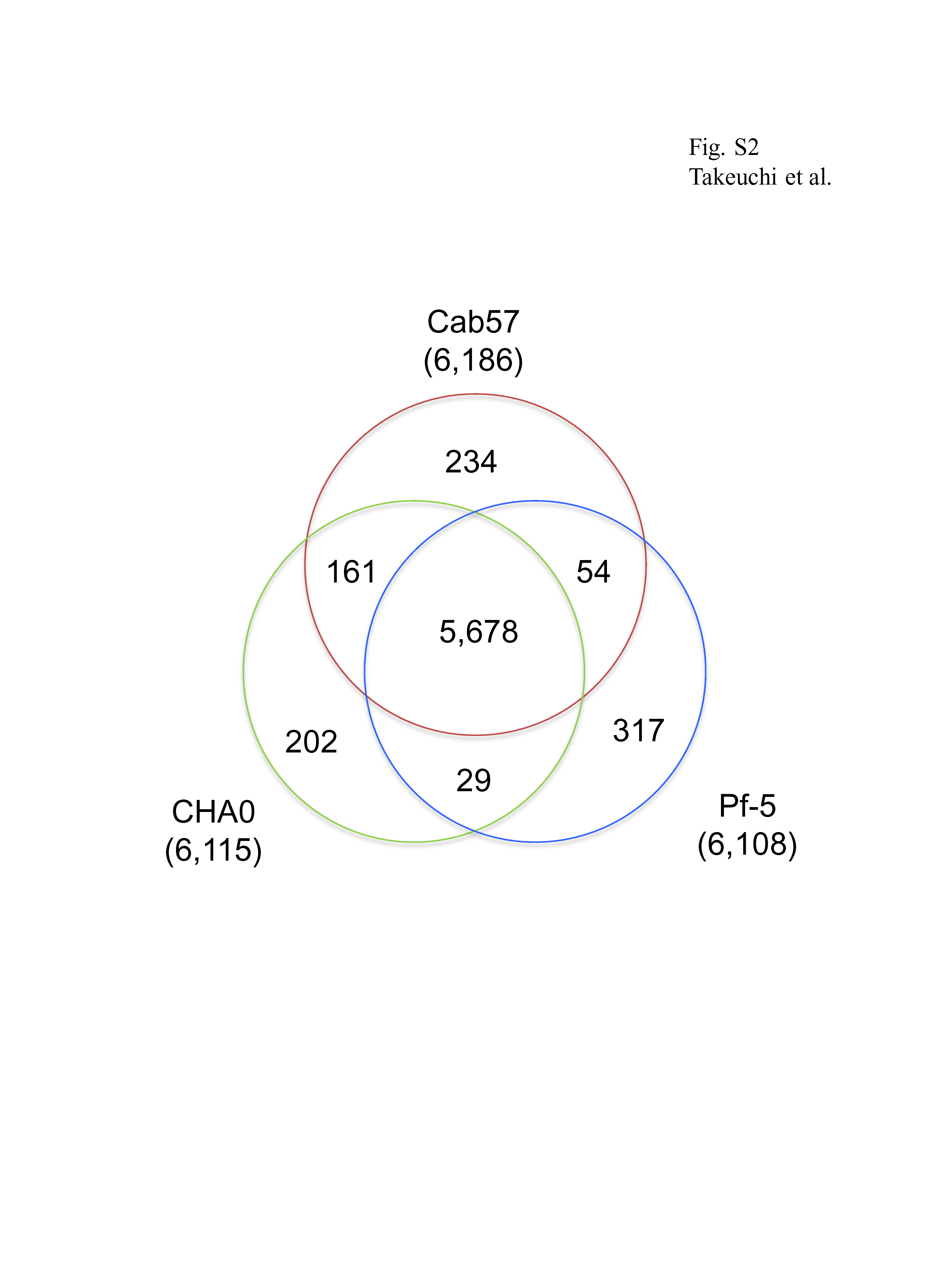

Supplement: Figure S2 — Venn diagram comparing the coding sequence sets of P. protegens strains Cab57, CHA0, and Pf-5. The number of orthologous coding sequences (CDSs) shared by all strains is in the center. The numbers in the non-overlapping portions of each circle represent the number of CDSs unique to each strain. The total number of CDSs within each genome is listed below the strain name. Each set of genes was compared to the other two sets using BLASTp, and sequence identity cut-off was set at 60% to identify common genes. Note that due to multiple hits, gene numbers did not add up to the strain totals. (TIF) [file pone.0093683.s002.tif]

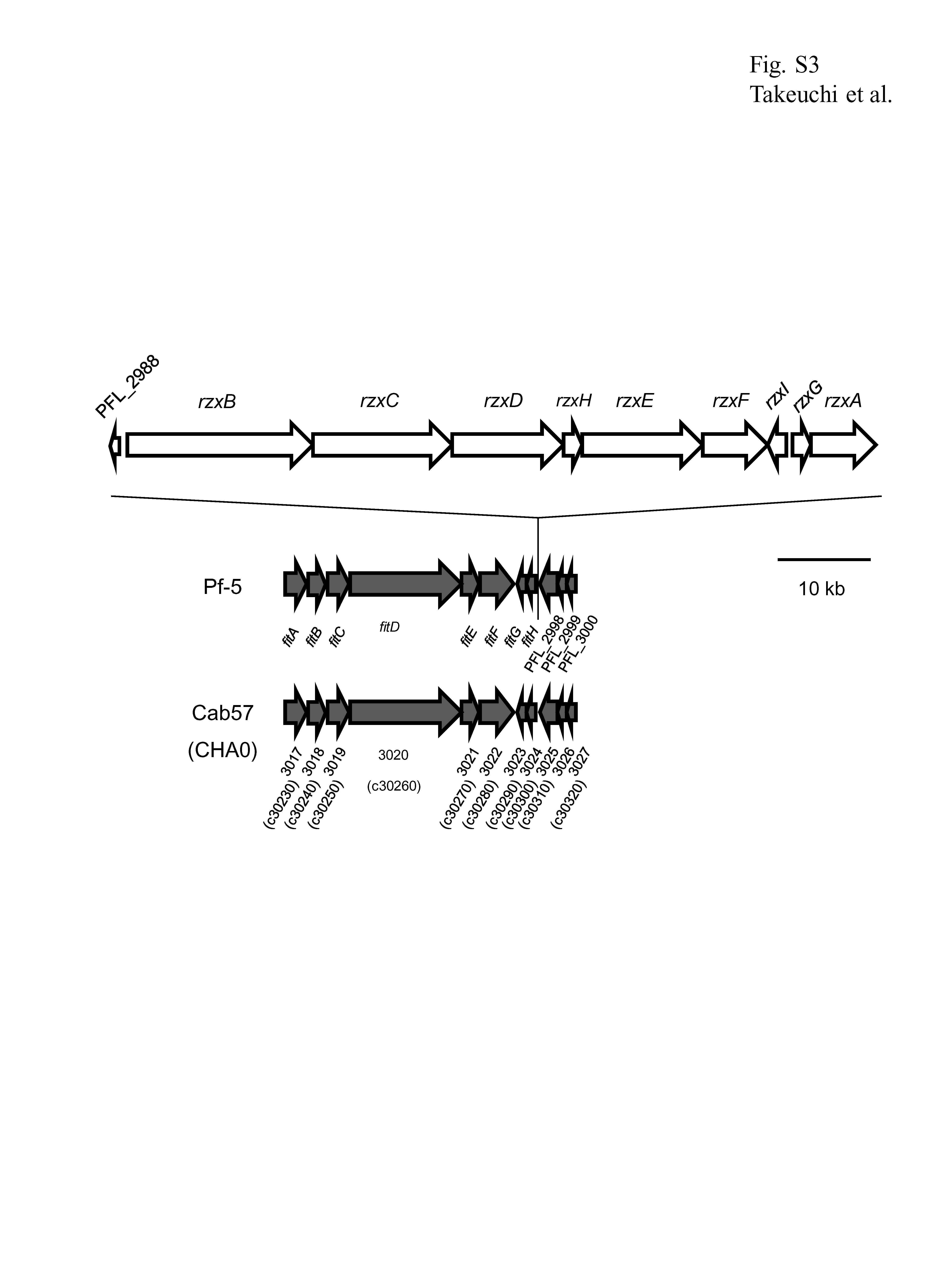

Supplement: Figure S3 — Genetic organization of fit cluster (for FitD toxin) and rzx cluster (for rhizoxin analogs) in P. protegens Pf-5 and the corresponding regions in P. protegens Cab57 and CHA0. The conserved genes are colored gray, and strain-specific genes are colored white. (TIF) [file pone.0093683.s003.tif]

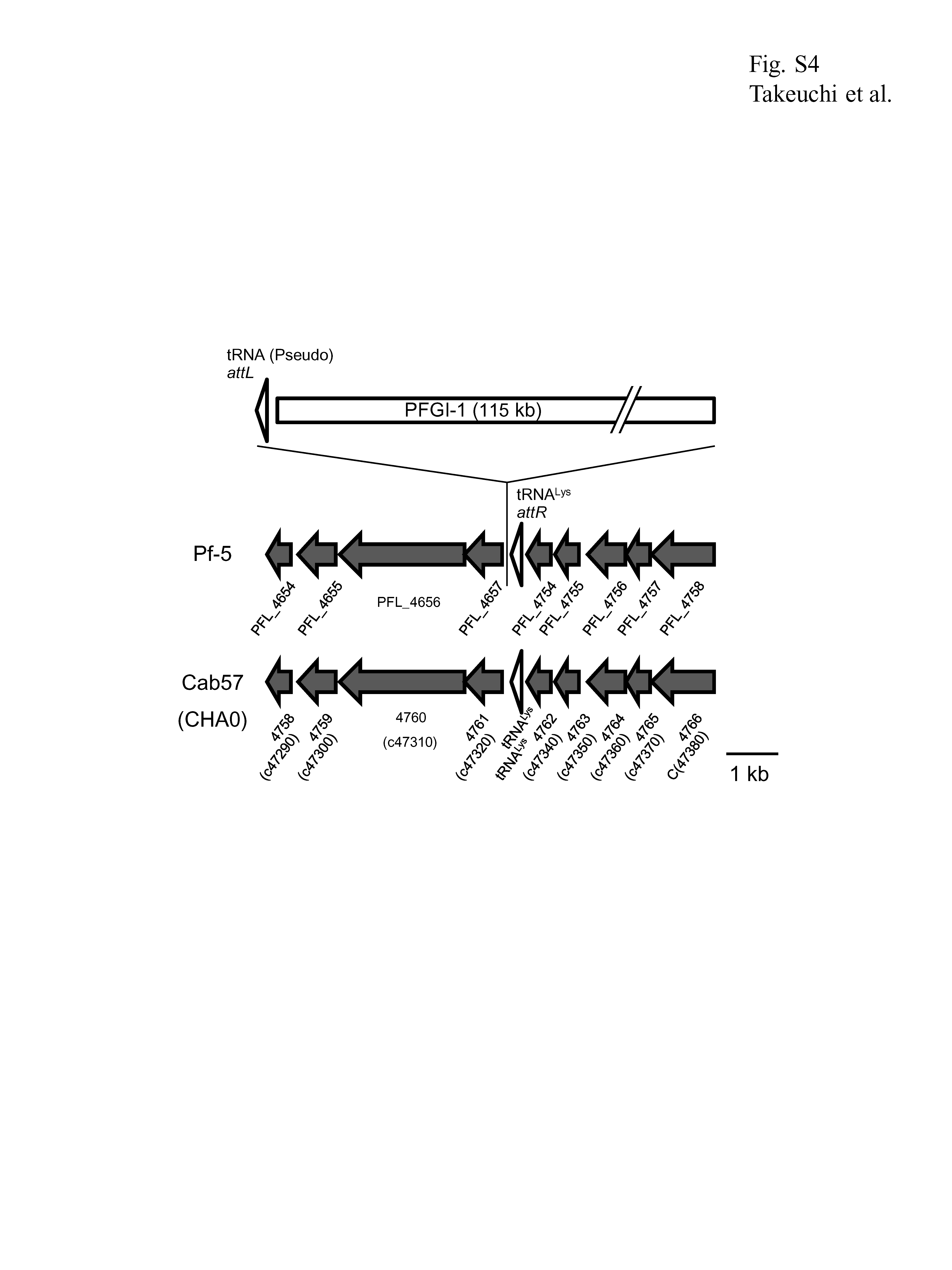

Supplement: Figure S4 — Genetic organization of the region surrounding the genomic island PFGI-1 in P. protegens Pf-5 and the corresponding regions in P. protegens Cab57 and CHA0. The conserved genes are colored gray, and strain-specific genes are colored white. (TIF) [file pone.0093683.s004.tif]

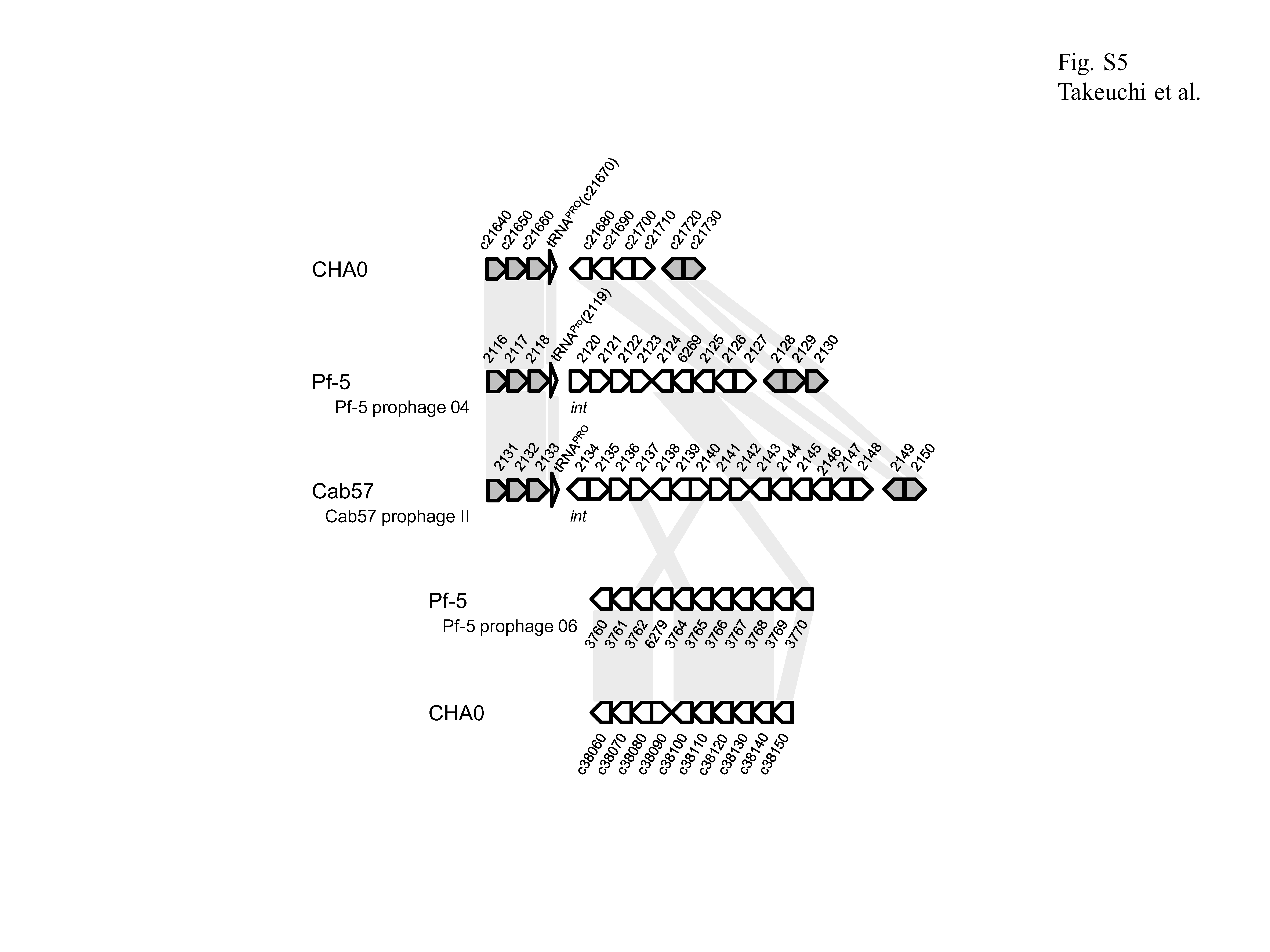

Supplement: Figure S5 — Genetic organization of the region surrounding Prophage II in P. protegens Cab57 and the corresponding regions in P. protegens Pf-5 and CHA0. Homologous genes are connected with gray shading. The genes in prophage are colored white and genes outside prophage are colored gray. The sizes of genes are not to scale. (TIF) [file pone.0093683.s005.tif]

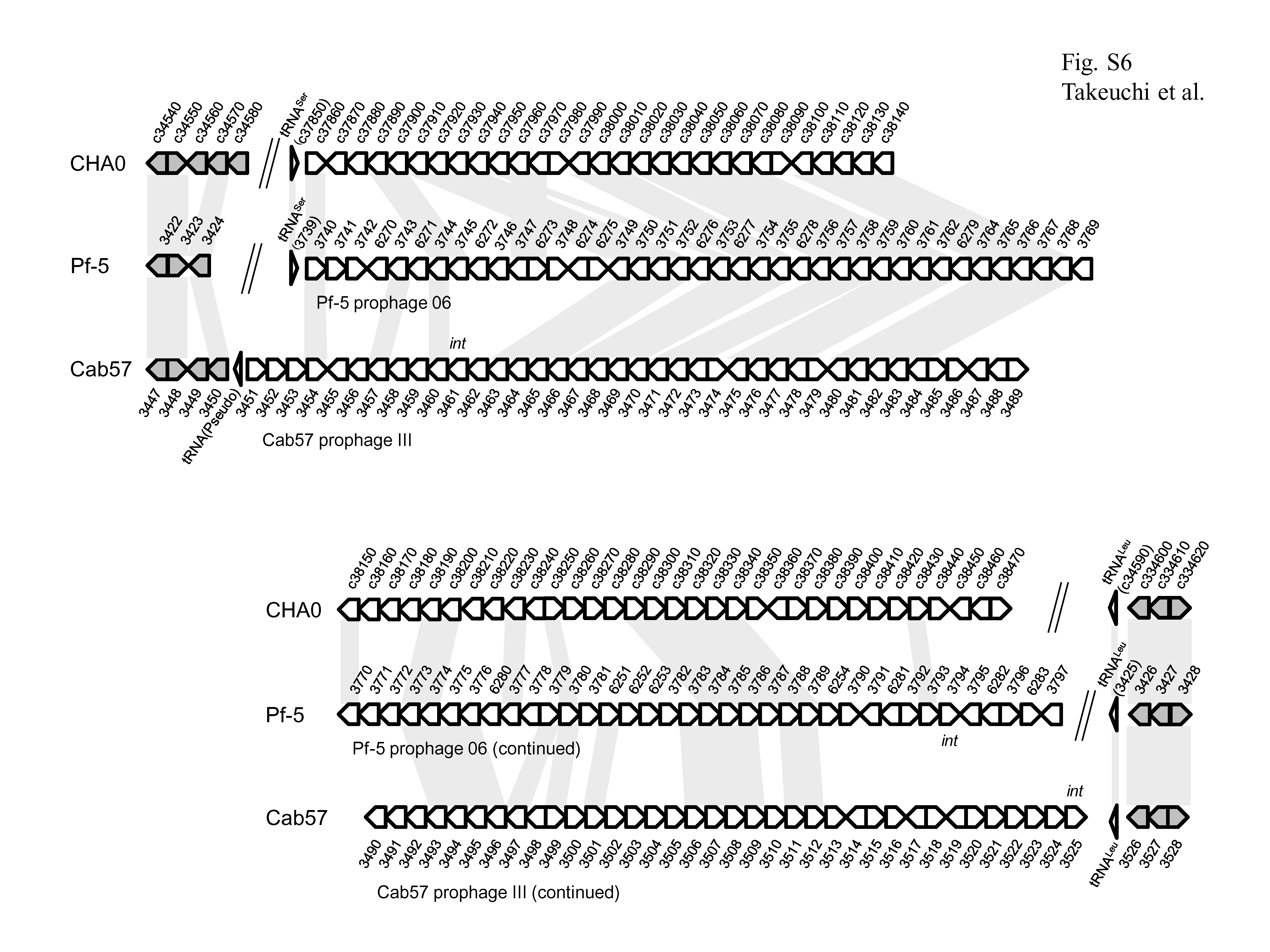

Supplement: Figure S6 — Genetic organization of the region surrounding Prophage III in P. protegens Cab57 and the corresponding regions in P. protegens Pf-5 and CHA0. Homologous genes are connected with gray shading. The genes in prophage are colored white and genes outside prophage are colored gray. The sizes of genes are not to scale. (TIF) [file pone.0093683.s006.tif]

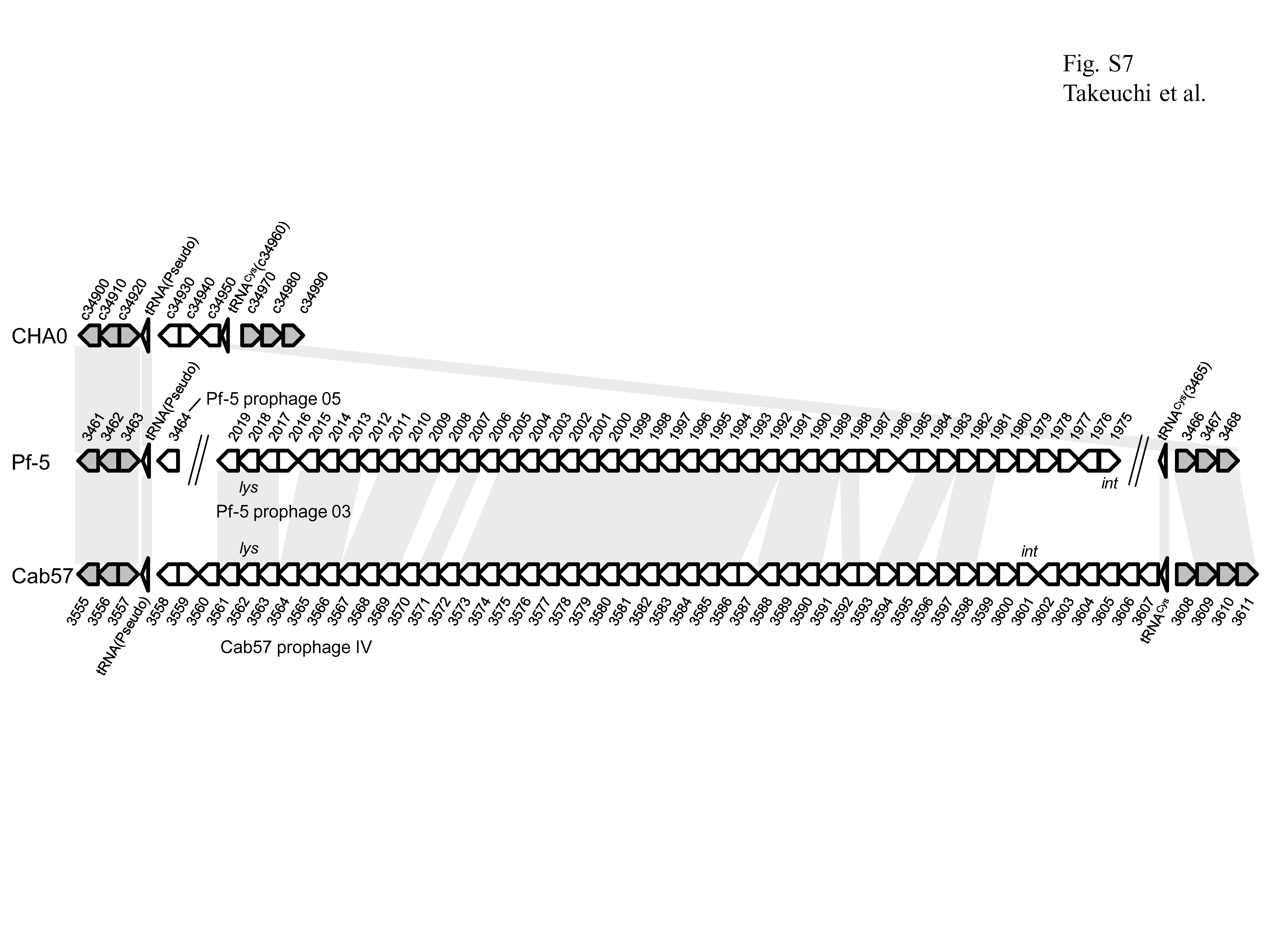

Supplement: Figure S7 — Genetic organization of the region surrounding Prophage IV in P. protegens Cab57 and the corresponding regions in P. protegens Pf-5 and CHA0. Homologous genes are connected with gray shading. The genes in prophage are colored white and genes outside prophage are colored gray. The sizes of genes are not to scale. (TIF) [file pone.0093683.s007.tif]

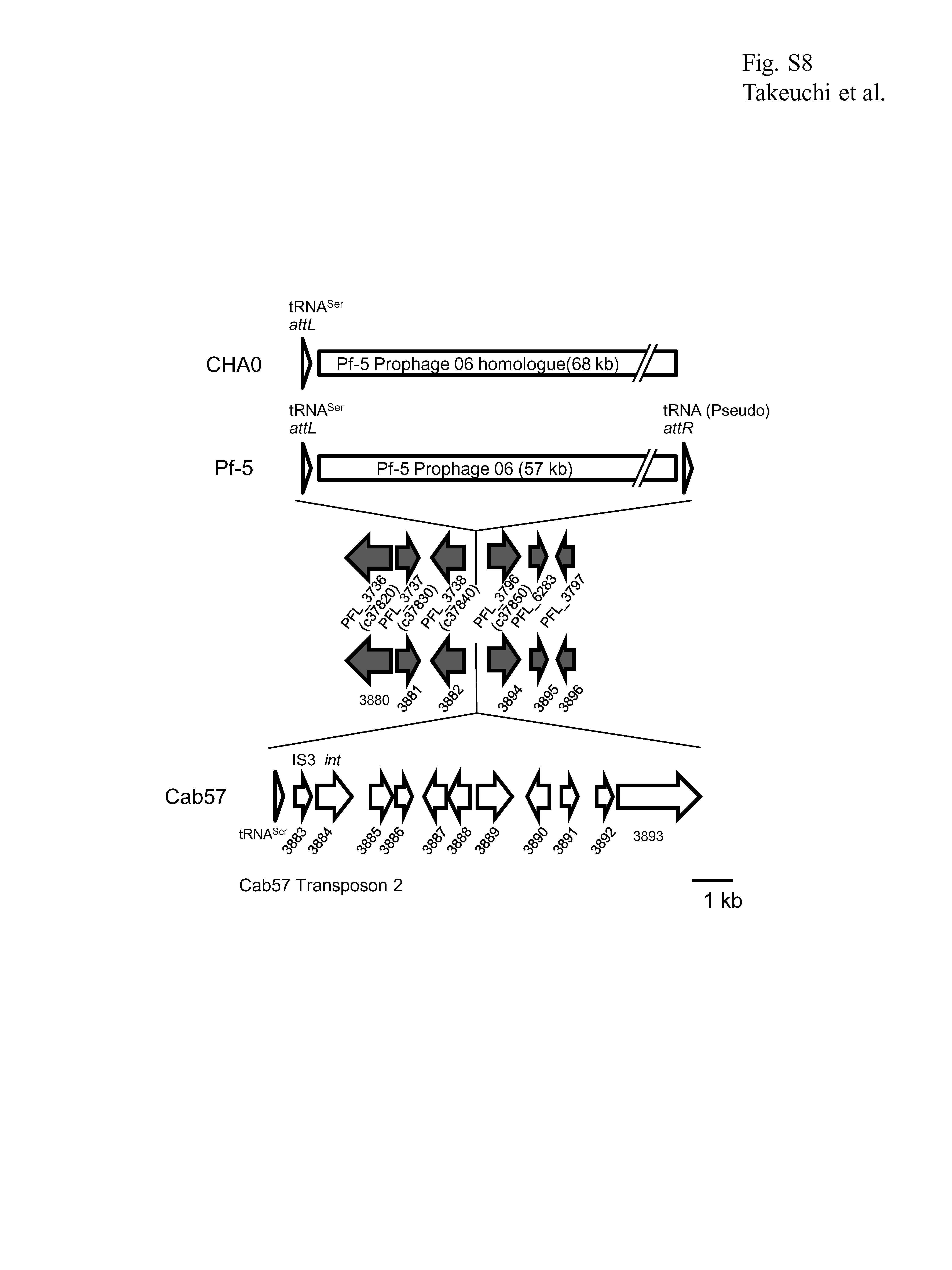

Supplement: Figure S8 — Genetic organization of the region surrounding Transposon 2 in P. protegens Cab57 and the corresponding regions in P. protegens Pf-5 and CHA0. The conserved genes are colored gray, and strain-specific genes are colored white. (TIF) [file pone.0093683.s008.tif]
