# Supplementary material for: Complete Genome Sequence of the Biocontrol Strain Pseudomonas protegens Cab57 Discovered in Japan Reveals Strain-Specific Diversity of This Species
Source: PLoS One. 2014 Apr 2;9(4):e93683. doi: 10.1371/journal.pone.0093683 (PMC3973561; doi:10.1371/journal.pone.0093683)
Supplement: Table S1 — Sequence analysis of gene clusters for the synthesis of antibiotics and exoenzyme in P. protegens Cab57 and similarities to those in P. protegens Pf-5. (DOCX) [file pone.0093683.s009.docx]

**Table S1.**

Sequence analysis of gene clusters for the synthesis of antibiotics and exoenzyme in *P. protegens* Cab57 and similarities to those in *P. protegens* Pf-5.

| Gene ID | Gene name | Position | Size of product  (amino acids) | % amino acid  homology |
| --- | --- | --- | --- | --- |
| *hcn* cluster (for hydrogen cyanide) | | | | |
| 2622 | *hcnA* | 2876481..2876798 | 105 | 100 |
| 2623 | *hcnB* | 2876795..2878204 | 469 | 100 |
| 2624 | *hcnC* | 2878197..2879450 | 417 | 99.5 |
|  |  |  |  |  |
| *plt* cluster (for hydrogen cyanide) | | |  | |
| 2829 | *pltM* | 3104673..3106181 | 502 | 99.6 |
| 2830 | *pltR* | 3106178..3106720 | 180 | 99.4 |
| 2831 | *pltL* | 3107695..3107961 | 88 | 100 |
| 2832 | *pltA* | 3107975..3109324 | 449 | 100 |
| 2833 | *pltB* | 3109357..3116733 | 2458 | 99.4 |
| 2834 | *pltC* | 3116782..3122106 | 1774 | 99.0 |
| 2835 | *pltD* | 3122157..3123791 | 544 | 99.3 |
| 2836 | *pltE* | 3123793..3124935 | 380 | 100 |
| 2837 | *pltF* | 3124932..3126428 | 498 | 99.0 |
| 2838 | *pltG* | 3126432..3127214 | 260 | 99.6 |
| 2839 | *pltZ* | 3127220..3127855 | 211 | 99.5 |
| 2840 | *pltI* | 3127967..3128980 | 337 | 99.4 |
| 2841 | *pltJ* | 3128977..3130746 | 589 | 100 |
| 2842 | *pltK* | 3130834..3131898 | 354 | 99.4 |
| 2843 | *pltN* | 3131903..3133021 | 372 | 100 |
| 2844 | *pltO* | 3133033..3134529 | 498 | 98.8 |
| 2845 | *pltP* | 3134601..3135200 | 199 | 99.5 |
|  |  |  |  |  |
| *prn* cluster (for pyrrolnitrin) | | |  | |
| 3743 | *prnA* | 4135440..4137053 | 537 | 99.3 |
| 3744 | *prnB* | 4137053..4138138 | 361 | 98.6 |
| 3745 | *prnC* | 4138181..4139884 | 567 | 99.7 |
| 3746 | *prnD* | 4139909..4141000 | 363 | 99.5 |
|  |  |  |  |  |
| *phl* cluster (for 2,4-diacetylphloroglucinol) | | | | |
| 5898 | *phlH* | 6517190..6517861 | 223 | 99.6 |
| 5899 | *phlG* | 6518003..6518887 | 294 | 98.3 |
| 5900 | *phlF* | 6518939..6519541 | 200 | 99.0 |
| 5901 | *phlA* | 6520003..6521091 | 360 | 99.2 |
| 5902 | *phlC* | 6521121..6522317 | 398 | 99.5 |
| 5903 | *phlB* | 6522330..6522770 | 146 | 96.6 |
| 5904 | *phlD* | 6523005..6524027 | 340 | 100 |
| 5905 | *phlE* | 6524137..6525402 | 421 | 99.3 |
|  |  |  |  |  |
| *apr* cluster | | | | |
| 3232 | *aprF* | 3593780..3595129 | 449 | 99.6 |
| 3233 | *aprE* | 3595144..3596478 | 438 | 99.8 |
| 3234 | *aprD* | 3596475..3598256 | 593 | 99.7 |
| 3236 | *aprA* | 3598946..3600394 | 482 | 99.8 |
