# Supplementary material for: Complete Genome Sequence of the Biocontrol Strain Pseudomonas protegens Cab57 Discovered in Japan Reveals Strain-Specific Diversity of This Species
Source: PLoS One. 2014 Apr 2;9(4):e93683. doi: 10.1371/journal.pone.0093683 (PMC3973561; doi:10.1371/journal.pone.0093683)
Supplement: Table S2 — Sequence analysis of Gac/Rsm homologues in P. protegens Cab57 and similarities to those in P. protegens Pf-5. (DOCX) [file pone.0093683.s010.docx]

**Table S2.**

Sequence analysis of Gac/Rsm homologues in *P. protegens* Cab57 and similarities to those in *P. protegens* Pf-5.

| Gene ID | Gene name | Position | | Size of product | | % homology |
| --- | --- | --- | --- | --- | --- | --- |
|  | | | (amino acids) | | (amino acids) | |
| 4560 | *gacS* | 5093736..5096489 | | 917 | | 100 |
| 3701 | *gacA* | 4089669..4090253 | | 194 | | 100 |
| 4608 | *rsmA* | 5146919..5147107 | | 62 | | 100 |
| 2108 | *rsmE* | 2325113..2325307 | | 64 | | 100 |
| 0678 | *retS* | 769346.. 772132 | | 928 | | 99.8 |
| 5395 | *ladS* | 5976609..5979020 | | 803 | | 99.1 |
| small regulatory RNAs | | | (nucleotides) | | (nucleotides) | |
|  | *rsmZ* | 1398027..1398153 | | 127 | | 100 |
|  | *rsmY* | 6232077..6232194 | | 118 | | 100 |
|  | *rsmX* | 4700464..4700582 | | 119 | | 100 |
