# Supplementary material for: Complete Genome Sequence of the Biocontrol Strain Pseudomonas protegens Cab57 Discovered in Japan Reveals Strain-Specific Diversity of This Species
Source: PLoS One. 2014 Apr 2;9(4):e93683. doi: 10.1371/journal.pone.0093683 (PMC3973561; doi:10.1371/journal.pone.0093683)
Supplement: Table S3 — Sequence analysis of gene clusters for the synthesis of cyclic lipopeptide, siderophores, and toxin in P. protegens Cab57 and similarities to those in P. protegens Pf-5. (DOCX) [file pone.0093683.s011.docx]

**Table S3.**

Sequence analysis of gene clusters for the synthesis of cyclic lipopeptide, siderophores, and toxin in *P. protegens* Cab57 and similarities to those in *P. protegens* Pf-5.

| Gene ID | Gene name  (ID for PFL) | Position | Size of product  (amino acids) | % amino acid  homology |
| --- | --- | --- | --- | --- |
| *pvd* cluster (for pyoverdine) | | |  | |
| 2934 | *pvdQ* (2902) | 3235469..3237802 | 777 | 99.8 |
| 2935 | *fpvR* (2903) | 3237853..3238866 | 337 | 100 |
| 4179 | *pvdA* (4079) | 4632960..4634297 | 445 | 99.8 |
| 4180 | *fpvI* | 4634447..4634992 | 181 | 100 |
| 4181 | RND efflux  Transporter  (4081) | 4635256..4636428 | 390 | 99.7 |
| 4182 | ABC efflux  Transporter  (4082) | 4636429..4638402 | 657 | 99.4 |
| 4183 | RND efflux  Transporter  (4083) | 4638410..4639801 | 463 | 97.6 |
| 4186 | *pvdP* (4086) | 4640668..4642272 | 534 | 99.4 |
| 4187 | *pvdM* | 4642504..4643877 | 457 | 98.7 |
| 4188 | *pvdN* | 4643938..4645194 | 418 | 99.8 |
| 4189 | *pvdO* | 4645242..4646135 | 297 | 100 |
| 4190 | *pvdF* | 4646190..4647035 | 281 | 100 |
| 4191 | *pvdE* | 4647254..4648906 | 550 | 99.3 |
| 4192 | *fpvA* | 4649002..4651473 | 823 | 98.3 |
| 4193 | *pvdD* | 4651864..4664286 | 4140 | 98.5 |
| 4195 | *pvdJ* | 4665109..4671696 | 2195 | 97.4 |
| 4196 | *pvdI* (4095) | 4671696..4682843 | 3715 | 99.3 |
| 4197 | Siderophore-  Interacting protein  (4096) | 4683056..4684024 | 322 | 99.4 |
| 4198 | PFL_4097 | 4684206..4684946 | 246 | 100 |
| 4266 | PFL_4169 | 4769834..4771057 | 407 | 98.5 |
| 4267 | PFL_4170 | 4771054..4771596 | 180 | 97.2 |
| 4268 | PFL_4171 | 4771593..4771931 | 112 | 97.3 |
| 4269 | PFL_4172 | 4771928..4772500 | 190 | 99.5 |
| 4270 | PFL_4173 | 4772536..4773465 | 309 | 99.4 |
| 4271 | PFL_4174 | 4773462..4774205 | 247 | 98.0 |
| 4272 | PFL_4175 | 4774205..4775104 | 299 | 99.7 |
| 4273 | PFL_4176 | 4775105..4776088 | 327 | 100 |
| 4275 | PFL_4178 | 4777551..4777775 | 74 | 100 |
| 4276 | *pvdH* (4179) | 4777858..4779270 | 470 | 98.7 |
| 4286 | *pvdL* (4189) | 4789512..4802528 | 4338 | 97.7 |
| 4287 | *pvdS* | 4802902..4803450 | 182 | 100 |
| 4288 | *pvdY* (4191) | 4803484..4803843 | 119 | 98.3 |
|  |  |  |  |  |
| *pch cluster* (for enantio-pyochelin) | | | | |
| 3642 | *pchA* | 4010132..4011565 | 477 | 99.1 |
| 3643 | *pchB* | 4011558..4011926 | 122 | 100 |
| 3644 | *pchC* | 4011905..4012684 | 259 | 99.2 |
| 3645 | *pchK* | 4012681..4013781 | 366 | 99.5 |
| 3646 | *pchF* | 4013793..4019216 | 1807 | 99.2 |
| 3647 | *pchE* | 4019213..4022683 | 1156 | 99.3 |
| 3648 | *pchI* | 4022673..4024439 | 588 | 99.7 |
| 3649 | *pchH* | 4024436..4026151 | 577 | 99.0 |
| 3650 | *pchD* | 4026174..4027841 | 555 | 99.1 |
| 3651 | *pchR* | 4028216..4029118 | 300 | 100 |
|  |  |  |  |  |
| *ofa* cluster (for orfamide A) | | | | |
| 2162 | *ofaA* | 2374352..2380717 | 2121 | 99.0 |
| 2163 | *ofaB* | 2381035..2394138 | 4367 | 99.2 |
| 2164 | *ofaC* | 2394135..2408840 | 4901 | 99.7 |
|  |  |  |  |  |
| *fit* cluster (for FitD toxin) | | | | |
| 3017 | *fitA* | 3336328..3338415 | 695 | 99.7 |
| 3018 | *fitB* | 3338412..3339800 | 462 | 99.6 |
| 3019 | *fitC* | 3339845..3341962 | 705 | 100 |
| 3020 | *fitD* | 3342084..3351083 | 2999 | 97.5 |
| 3021 | *fitE* | 3351160..3352629 | 489 | 94.8 |
| 3022 | *fitF* | 3352697..3355936 | 1079 | 95.0 |
| 3023 | *fitG* | 3355933..3356850 | 305 | 98.4 |
| 3024 | *fitH* | 3356871..3357851 | 326 | 98.2 |
