# Supplementary material for: Complete Genome Sequence of the Biocontrol Strain Pseudomonas protegens Cab57 Discovered in Japan Reveals Strain-Specific Diversity of This Species
Source: PLoS One. 2014 Apr 2;9(4):e93683. doi: 10.1371/journal.pone.0093683 (PMC3973561; doi:10.1371/journal.pone.0093683)
Supplement: Table S4 — Sequence analysis of the transposon of the P. protegens Cab57 genome. (DOCX) [file pone.0093683.s012.docx]

**Table S4.**

Sequence analysis of the transposon of the *P. protegens* Cab57 genome.

| Gene  ID | Position | Size of  product  (aa) | NCBI Ref. of the  closest protein,  blastp E-value | Predicted function  (region name) | | | Homologue in CHA0* |
| --- | --- | --- | --- | --- | --- | --- | --- |
| Transposon 1 (1981-1998) | | | | | | |  |
| 1981 | 2176361..2177809 | 482 | YP_007110352 from  *Geitlerinema* sp. PCC  7407, 2.00E-37 | Mannose-6-phosphate  isomerase | | | c19670 |
| 1982 | 2177849..2178964 | 371 | YP_351415 from  *Pseudomonas fluorescens*  Pf0-1, 1.00E-137 | Group 1 glycosyl  transferase  (RfaG) | | | c19680 |
| 1983 | 2178977..2179720 | 247 | YP_468305 from  *Rhizobium etli* CFN 42,  1.00E-105 | UDP-hexose  transferase | | | c19690 |
| 1984 | 2179721..2180656 | 311 | YP_001789664 from  *Leptothrix cholodnii* SP-6,  1.00E-122 | NAD-dependent  epimerase/dehydratase | | | c19700 |
| 1985 | 2180664..2181749 | 361 | ZP_09868818 from  *Thiorhodovibrio* sp. 970,  1.00E-174 | GDP-mannose 4,6-  Dehydratase  (Gmd) | | | c19710 |
| 1986 | 2182596..2183606 | 336 | HRAG_02102 from  *Helicobacter bilis*  ATCC43879, 6.00E-29 | 3-isopropylmalate  dehydratase large  subunit | | | c19720 |
| 1987 | 2183599..2184942 | 447 | YP_303935 from  *Methanosarcina barkeri*  str. *Fusaro*, 1.00E-106 | ABC polysaccharide/  polyol phosphate  transport system,  ATPase component | | | c19730 |
| 1988 | 2184932..2185762 | 276 | ZP_18347591 from  *Pseudomonas fluorescens*  R124, 7.00E-96 | ABC polysaccharide/  polyol phosphate export  system, permease  component  (TagG) | | | - |
| 1989 | 2185759..2187702 | 647 | YP_935100 from  *Azoarcus* sp. BH72,  0.0 | Hypothetical protein  (RfaG) | | | c19740 |
| 1990 | 2187760..2188761 | 333 | YP_550792 from  *Polaromonas* sp. JS666,  1.00E-144 | NAD-dependent  epimerase/dehydratase | | | c19750 |
| 1991 | 2189649..2190380 | 243 | AZC_4004 from  *Azorhizobium caulinodans*  ORS 571, 2.00E-40 | O-antigen acetylase | c19760 | | |
| 1992 | 2191714..2192568 | 284 | YP_003368263 from  *Citrobacter rodentium*  ICC168, 1.00E-123 | ISEc14 transposase B | - | | |
| 1993 | 2192565..2192849 | 94 | YP_003368264 from  *Citrobacter rodentium*  ICC168, 1.00E-35 | ISEc14 transposase A | - | | |
| 1994 | 2193147..2194247 | 366 | AZA_38872 from  *Azospirillum amazonense*  Y2, 4.00E-12 | Hypothetical protein | c19780 | | |
| 1995 | 2195602..2195991 | 129 | YP_004512144 from  *Methylomonas methanica*  MC09, 2.00E-20 | Family 2 glycosyl  transferase | - | | |
| 1996 | 2195988..2196716 | 242 | BRAO375_600014 from  *Bradyrhizobium* sp. ORS  375, 6.00E-72 | Conserved hypothetical  protein;putative S-  adenosyl-L-methionine  (SAM)-dependent  methyltransferase | c19790 | | |
| 1997 | 2196713..2197690 | 325 | YP_003546403 from  *Sphingobium japonicum*  UT26S, 1.00E-114 | Dolichol-phosphate  mannosyltransferase | c19800 | | |
| 1998 | 2197730..2198710 | 326 | Mettu_3507 from  *Methylobacter*  *Tundripaludum* SV96,  1.00E-102 | UDP-glucose 4-  epimerase | c19810 | | |
|  |  |  |  |  |  | | |
| Transposon 2 (3883-3893) | | | | | | | |
| 3883 | 4318849..4319142 | 97 | YP_001351351 from  *Pseudomonas aeruginosa*  PA7, 3.00E-49 | Transposase IS3 | - | | |
| 3884 | 4319139..4320005 | 288 | ZP_04937914 from  *Pseudomonas aeruginosa*  2192, 1.00E-160 | Integrase, catalytic  domain | - | | |
| 3885 | 4320606..4321178 | 190 | PseBG33_4864 from  *Pseudomonas synxantha*  BG33R, 6.00E-32 | Hypothetical protein | - | | |
| 3886 | 4321189..4321626 | 145 | PMI25_04718 from  *Pseudomonas* sp. GM30,  6.00E-53 | Hypothetical protein | c38440 | | |
| 3887 | 4322010..4322528 | 172 | ZP_21125839 from  *Pseudomonas syringae* pv.  *syringae* B64, 3.00E-48 | Bacteriophage host  lysis protein | c38450 | | |
| 3888 | 4322525..4322989 | 154 | NP_793172 from  *Pseudomonas syringae* pv.  *tomato* str. DC3000,  1.00E-59 | Lysozyme | c38460 | | |
| 3889 | 4323131..4323907 | 258 | YP_260900 from  *Pseudomonas fluorescens*  (strain Pf-5 / ATCC BAA-  477), 1.00E-147 | Hypothetical protein | c38470 | | |
| 3890 | 4324281..4324712 | 143 | CUS_5106 from  *Ruminococcus albus* 8,  0.83 | Response regulator,  receiver domain  protein | - | | |
| 3891 | 4325144..4325380 | 78 | CSV86_27122 from  *Pseudomonas putida*  CSV86, 5.00E-20 | Phosphoenolpyruvate  carboxykinase | - | | |
| 3892 | 4326201..4326527 | 108 | PflSS101_2102 from  *Pseudomonas fluorescens*  SS101, 1.00E-47 | Altronate dehydratase | c38480 | | |
| 3893 | 4326562..4328514 | 650 | ZP_17665662 from  *Pseudomonas fluorescens*  SS101, 0.0 | D-arabinono-1,4-  lactone oxidase  domain protein | | c38490 | |

*The “-” refers to the absence of the homologue in CHA0.
