# Supplementary material for: Complete Genome Sequence of the Biocontrol Strain Pseudomonas protegens Cab57 Discovered in Japan Reveals Strain-Specific Diversity of This Species
Source: PLoS One. 2014 Apr 2;9(4):e93683. doi: 10.1371/journal.pone.0093683 (PMC3973561; doi:10.1371/journal.pone.0093683)
Supplement: Table S5 — Sequence analysis of gene clusters in P. protegens Cab57 which are absent from Pf-5 genome. (DOCX) [file pone.0093683.s013.docx]

**Table S5.**

Sequence analysis of gene clusters in *P. protegens* Cab57 which are absent from Pf-5 genome.

| Gene  ID | Position | Size of  product  (aa) | NCBI Ref. of the  closest protein,  blastp E-value | Predicted function  (region name) | Homologue in CHA0* |
| --- | --- | --- | --- | --- | --- |
| Cluster S1 (0832-0846) | | | | | |
| 0832 | 935424..935876 | 150 | PMI20_00165 from  *Pseudomonas*sp. GM17,  6.0E-38 | Alpha-mannosidase | c08220 |
| 0833 | 936134..936697 | 187 | ZP_10173831 from  *Pseudomonas chlororaphis*  O6, 2.0E-73 | Phosphoglycerate mutase | c08230 |
| 0834 | 936694..937731 | 345 | YP_007030361 from  *Pseudomonas* sp. UW4,  1.0E-167 | NAD-dependent  epimerase/dehydratase  (HpnA) | c08240 |
| 0835 | 937735..938154 | 139 | YP_007030360 from  *Pseudomonas* sp. UW4,  9.0E-58 | snoaL-like polyketide  cyclase | c08250 |
| 0836 | 938270..939151 | 293 | YP_007030359　from  *Pseudomonas* sp. UW4,  1.00E-143 | LysR family transcriptional  regulator | c08260 |
| 0837 | 939355..940233 | 292 | ZP_10848825 from  *Pseudomonas fragi* A22,  1.00E-161 | LysR family transcriptional  regulator | - |
| 0838 | 940363..941241 | 292 | ZP_06689128 from  *Achromobacter piechaudii*  ATCC 43553, 1.00E-123 | Poly(aspartic acid) hydrolase  (LpqC) | - |
| 0839 | 941368..942936 | 522 | ZP_10848823 from  *Pseudomonas fragi* A22,  0.0 | Extracellular solute-  binding protein | - |
| 0840 | 942948..943889 | 313 | ZP_06689126 from  *Achromobacter piechaudii*  ATCC 43553, 1.00E-130 | Dipeptide ABC superfamily  ATP binding cassette  transporter, permease protein  (DppB) | - |
| 0841 | 943886..944755 | 289 | ZP_10180755 from  *Microvirga* sp. WSM3557,  3.00E-99 | ABC-type dipeptide/  oligopeptide/nickel transport  system, permease component  (DppC) | - |
| 0842 | 944758..946389 | 543 | ZP_10848820 from  *Pseudomonas fragi* A22,  0.0 | Peptide ABC transporter  nucleotide binding/ATPase  protein | - |
| 0843 | 946518..947798 | 426 | ZP_10848819 from  *Pseudomonas fragi* A22,  0.0 | Porin, OprD | - |
| 0844 | 947866..949380 | 504 | ZP_10848818 from  *Pseudomonas fragi* A22,  0.0 | 6-aminohexanoate-dimer  hydrolase  (Beta-lactamase)  (AmpC) | - |
| 0845 | 949377..950702 | 441 | ZP_10848817 from  *Pseudomonas fragi* A22,  0.0 | beta-alanine-pyruvate  transaminase | - |
| 0846 | 950717..952135 | 472 | ZP_10848816 from  *Pseudomonas fragi* A22,  0.0 | Aldehyde dehydrogenase | - |
|  |  |  |  |  |  |
| Cluster S2 (1921-1928) | | | | | |
| 1921 | 2101106..2101858 | 250 | ZP_05640304 from  *Pseudomonas syringae* pv.  *tabaci* str. ATCC11528,  1.00E-110 | Uroporphyrin-III  C-methyltransferase  (SUMT) | c19090 |
| 1922 | 2101868..2104585 | 905 | ZP_05640303 from  *Pseudomonas syringae* pv.  *tabaci* str. ATCC11528,  1.00E-164 | Nitrate reductase | c19100 |
| 1923 | 2104585..2104902 | 105 | ZP_10176329 from  *Pseudomonas chlororaphis*  O6, 2.00E-50 | Assimilatory nitrite reductase  [NAD(P)H], small subunit | c19110 |
| 1924 | 2104899..2107352 | 817 | YP_002945411 from  *Variovorax paradoxus* S110  0.0 | Nitrite reductase (NAD(P)H),  large subunit  (NirB) | c19120 |
| 1925 | 2107743..2109413 | 556 | ZP_16686506 from  *Pseudomonas syringae* pv.  *japonica* str. M301072,  1.00E-160 | Protein kinase  (PP2Cc) | c19130 |
| 1926 | 2109421..2110632 | 403 | ZP_10176725 from  *Pseudomonas chlororaphis*  O6, 0.0 | Nitrite transporter  (NarK) | c19140 |
| 1927 | 2110977..2111552 | 191 | ZP_05640288 from  *Pseudomonas syringae* pv.  *tabaci* str. ATCC11528,  3.00E-96 | Response regulator  receiver: ANTAR  (AmiR) | c19150 |
| 1928 | 2111568..2112779 | 403 | YP_001188068 from  *Pseudomonas mendocina*  ymp, 1.0E-169 | Nitrate/sulfonate/  bicarbonate ABC  transporter periplasmic  component-like protein | c19160 |
|  |  |  |  |  |  |
| Cluster S3 (2197-2203) | | | | | |
| 2197 | 2442942..2443445 | 167 | ZP_10994713 from  *Pseudomonas fuscovaginae*  UPB0736, 8.00E-70 | Phage immunity protein;  membrane protein | - |
| 2198 | 2443816..2444685 | 289 | YP_004355159 from  *Pseudomonas brassicacearum*  subsp. *brassicacearum*  NFM421, 1.00E-136 | AraC family transcriptional  regulator | - |
| 2199 | 2444815..2445726 | 303 | YP_260014 from  *Pseudomonas protegens* Pf-5,  6.00E-84 | Transcriptional regulator  (LysR)  (HTH_1) | - |
| 2200 | 2445921..2446223 | 100 | AAI_02436 from  *Pseudomonas viridiflava*  UASWS0038, 4.00E-08 | Membrane-bound lytic  murein　transglycosylase B | - |
| 2201 | 2446378..2448003 | 541 | ZP_16387497 from  *Pseudomonas avellanae*  BPIC 631, 1.00E-129 | Penicillin-binding protein  (Ftsl) | - |
| 2202 | 2448027..2449937 | 636 | YP_608096 from  *Pseudomonas entomophila*  L48, 0.0 | Penicillin-binding protein 2 | c42010 |
| 2203 | 2450069..2452417 | 782 | YP_007027356 from  *Pseudomonas* sp. UW4,  0.0 | Penicillin-binding protein 1A  (MrcA) | - |
|  |  |  |  |  |  |
| Cluster S4 (2207-2210) | | | | | |
| 2207 | 2455772..2456206 | 144 | YP_005435396 from  *Rubrivivax gelatinosus*  IL144, 3.00E-44 | Lipoprotein | c22280 |
| 2208 | 2456239..2456607 | 122 | ZP_10142633 from  *Pseudomonas synxantha*  BG33R, 2.00E-57 | Antibiotic biosynthesis  monooxygenase domain  protein  (ABM) | c22290 |
| 2209 | 2456740..2457168 | 142 | NP_519972 from  *Ralstonia solanacearum*  GMI1000, 5.00E-48 | Transcription  regulator protein | c22300 |
| 2210 | 2457244..2457408 | 54 | ZP_10176165 from  *Pseudomonas chlororaphis*  O6, 7.00E-22 | Hypothetical protein | c22310 |
|  |  |  |  |  |  |
| Cluster S5 (2220-2233) | | | | | |
| 2220 | 2464525..2464926 | 133 | ZP_10176165 from  *Pseudomonas chlororaphis*  O6, 2.00E-67 | Hypothetical protein | c22410 |
| 2221 | 2464962..2465144 | 60 | ZP_18345479 from  *Pseudomonas fluorescens*  R124, 3.00E-17 | Periplasmic or secreted  lipoprotein　YcfA | c22420 |
| 2222 | 2465155..2465388 | 77 | LELG_02614 from  *Lodderomyces elongisporus*  NRRL YB-4239, 1.00E+00 | CCR4-NOT transcriptional  regulation complex, NOT5  subunit | c22430 |
| 2223 | 2465837..2466355 | 172 | NP_901925 from  *Chromobacterium violaceum*  ATCC 12472, 5.00E-64 | Maltose O-acetyltransferase | - |
| 2224 | 2466376..2467614 | 412 | NP_901926 from  *Chromobacterium violaceum*  ATCC 12472, 0.0 | Acetylornithine amino-  transferase　(BioA) | c22450 |
| 2225 | 2467592..2469379 | 595 | NP_901927 from  *Chromobacterium violaceum*  ATCC 12472, 0.0 | Transketolase | c22460 |
| 2226 | 2469476..2470321 | 281 | NP_901928 from  *Chromobacterium violaceum*  ATCC 12472, 1.00E-128 | Short-chain dehydrogenase  (fabG) | c22470 |
| 2227 | 2470314..2470937 | 207 | NP_901929 from  *Chromobacterium violaceum*  ATCC 12472, 5.00E-52 | Hypothetical protein | c22480 |
| 2228 | 2470944..2471729 | 261 | NP_901930 from  *Chromobacterium violaceum*  ATCC 12472, 1.00E-103 | Predicted hydrolases of the  HAD superfamily | c22490 |
| 2229 | 2471748..2472773 | 341 | NP_901931 from  *Chromobacterium violaceum*  ATCC 12472, 1.00E-130 | Acetylserotonin  O-methyltransferase | c22500 |
| 2230 | 2472883..2474073 | 396 | NP_901932 from  *Chromobacterium violaceum*  ATCC 12472, 1.00E-115 | Permeases of the major  facilitator superfamily | c22510 |
| 2231 | 2474427..2474708 | 93 | YP_259299 from  *Pseudomonas protegens* Pf-5,  5.00E-43 | Hypothetical protein | C22520 |
| 2232 | 2475504..2476382 | 292 | ZP_08732147 from  *Vibrio nigripulchritudo*  ATCC27043, 2.00E-65 | Hypothetical protein | - |
| 2233 | 2477110..2478003 | 297 | VMA_000067 from  *Vibrio mimicus* VM223,  9.00E-71 | Putative inner membrane  protein | - |
|  |  |  |  |  |  |
| Cluster S6 (2471-2474) | | | | | |
| 2471 | 2718831..2719700 | 289 | YP_007406296 from  *Serratia marcescens* WW4,  1.00E-129 | LysR family transcriptional  regulator | - |
| 2472 | 2719838..2720896 | 352 | ZP_18346257 from  *Pseudomonas fluoresce*ns  124, 1.00E-170 | Peptidylarginine deiminase | - |
| 2473 | 2720893..2721801 | 302 | YP_001081861 from  *Burkholderia mallei*  NCTC 10247, 1.00E-98 | Carbon-nitrogen family  hydrolase agmatine_aguB | - |
| 2474 | 2721803..2722924 | 373 | YP_348104 from  *Pseudomonas fluorescens*  f0-1, 1.00E-167 | Extracellular solute-binding  protein | - |
|  |  |  |  |  |  |
| Cluster S7 (2861-2864) | | | | | |
| 2861 | 3150192..3151085 | 297 | YP_765053 from  *Rhizobium leguminosarum*  bv. viciae 3841, 1.00E-105 | LysR family transcriptional  regulator | c28710 |
| 2862 | 3151182..3151502 | 106 | PMI26_03676 from  *Pseudomonas* sp. GM33,  4.00E-39 | Hydrolases of the alpha/  beta superfamily | - |
| 2863 | 3151517..3152728 | 403 | ZP_06834481 from  *Gluconacetobacter* *hansenii*  ATCC 23769, 1.00E-131 | Major facilitator superfamily  (MFS_1) | c28720 |
| 2864 | 3152906..3153094 | 62 | YP_259924 from  *Pseudomonas protegens* Pf-5,  2.00E-17 | Hypothetical protein | c28730 |
|  |  |  |  |  |  |
| Cluster S8 (3001-3007) | | | | | |
| 3001 | 3315694..3316035 | 113 | YP_004353898 from  *Pseudomonas brassicacearum*  subsp. *brassicacearum*  FM421, 4.00E-43 | Lipoprotein | c30110 |
| 3002 | 3316190..3317737 | 515 | ZP_06500372 from  *Pseudomonas syringae* pv.  *syringae* FF5, 0.0 | Deoxyribodipyrimidine  photolyase-like protein  (DPRP) | - |
| 3003 | 3317731..3317856 | 41 | I1A_002612 from  *Pseudomonas fluorescens*  R124, 8.00E-03 | Dehydrogenases with  Different　specificities  (related to short-chain  alcohol dehydrogenases) | - |
| 3004 | 3318137..3318367 | 76 | PMI35_06741 from  *Pseudomonas* sp. GM78,  3.00E-12 | Hypothetical protein | - |
| 3005 | 3318417..3318890 | 157 | PFLU_4286 from  *Pseudomonas fluorescen*s  SBW25, 7.00E-47 | DNA-directed RNA  polymerase, beta subunit/  140 kD subunit | - |
| 3006 | 3318887..3320347 | 486 | YP_003158736 from  *Desulfomicrobium baculatum*  DSM 4028, 1.00E-173 | Circadian clock protein KaiC | - |
| 3007 | 3320357..3321400 | 347 | PFLU_4284 from  *Pseudomonas fluorescens*  SBW25, 1.00E-115 | Signal transduction  histidine kinase | - |
|  |  |  |  |  |  |
| Cluster S9 (3036-3043) | | | | | |
| 3036 | 3371615..3372019 | 134 | BURK_015780 from  *Burkholderia* sp. SJ98,  4.00E-31 | Hypothetical protein | - |
| 3037 | 3372123..3372635 | 170 | YP_004948221 from  *Aggregatibacter*  *actinomycetemcomitans*  ANH9381, 5.00E-07 | ABC-type sugar transport  system, permease  component | - |
| 3038 | 3372922..3374559 | 545 | ZP_10143234 from  *Pseudomonas synxantha*  BG33R, 0.0 | Oxidoreductase, GMC  family | - |
| 3039 | 3374791..3375738 | 315 | ZP_10140951 from  *Pseudomonas synxantha*  BG33R, 1.00E-174 | Metallo-beta-lactamase  domain protein  (GloB) | - |
| 3040 | 3375786..3376658 | 290 | ZP_10141739 from  *Pseudomonas synxantha*  BG33R, 1.00E-151 | Transcriptional regulator,  LysR family | - |
| 3041 | 3376655..3377551 | 298 | ZP_10142899 from  *Pseudomonas synxantha*  BG33R, 1.00E-171 | alpha/beta hydrolase domain  protein  (Esterase_lipase) | - |
| 3042 | 3377600..3378529 | 309 | ZP_10143202 from  *Pseudomonas synxantha*  BG33R, 1.00E-167 | Transcriptional regulator,  LysR family | - |
| 3043 | 3379183..3379758 | 191 | PchlO6_3233 from  *Pseudomonas chlororaphis*  O6, 2.00E-90 | N-acetylglutamate synthase  (N-acetylornithine  aminotransferase) | - |
|  |  |  |  |  |  |
| Cluster S10 (3259-3262) | | | | | |
| 3259 | 3627773..3630823 | 1016 | YP_003003768 from  *Dickeya zeae* Ech1591,  0.0 | Formate dehydrogenase  subunit alpha | c32650 |
| 3260 | 3630837..3631718 | 293 | ZP_10486301 from  *Enterobacter radicincitans*  DSM 16656, 1.00E-130 | Formate dehydrogenase,  beta subunit | c32660 |
| 3261 | 3631718..3632350 | 210 | YP_002797618 from  *Azotobacter vinelandii* DJ,  3.00E-87 | Formate dehydrogenase  subunit gamma | c32670 |
| 3262 | 3632347..3633261 | 304 | ZP_11259135 from  *Pseudomonas* sp. HYS,  2.00E-99 | Formate dehydrogenase  accessory protein FdhE | c32680 |
|  |  |  |  |  |  |
| Cluster S11 (3624-3635) | | | | | |
| 3624 | 3987156..3988910 | 584 | YP_004476090 from  *Pseudomonas fulva* 12-X,  0.0 | Butyryl-CoA dehydrogenase | c35150 |
| 3625 | 3988971..3990212 | 413 | ZP_16731738 from  *Pseudomonas syringae* pv.  *aceris* str. M302273,  6.00E-74 | Thiolase | c35160 |
| 3626 | 3990209..3992107 | 632 | ZP_06492625 from  *Pseudomonas syringae* pv.  *syringae* FF5, 1.00E-123 | Feruloyl-CoA synthase  AMP-binding | c35170 |
| 3627 | 3992240..3993688 | 482 | YP_984052 from  *Polaromonas*  *aphthalenivorans* CJ2,  1.00E-162 | Aldehyde dehydrogenase | C35180 |
| 3628 | 3993794..3994624 | 276 | YP_001949689 from  *Burkholderia multivorans*  ATCC 17616, 1.00E-135 | p-hydroxycinnamoyl CoA  hydratase/lyase | c35190 |
| 3629 | 3994833..3995321 | 162 | ZP_16697677 from  *Pseudomonas syringae* pv.  *pisi* str. 1704B, 2.00E-31 | Regulatory protein, MarR | c35200 |
| 3630 | 3995376..3996698 | 440 | ZP_16685724 from  *Pseudomonas syringae* pv.  *japonica* str. M301072,  1.00E-150 | Outer membrane porin  (OprD) | c35210 |
| 3631 | 3997031..3998242 | 403 | ZP_17805146 from  *Pseudomonas syringae* pv.  *avellanae* str. ISPaVe013,  0.0 | Major facilitator family  transporter | c35220 |
| 3632 | 3998325..4000724 | 799 | ZP_16484792 from  *Pseudomonas syringae* pv.  *glycinea* str. race 4, 0.0 | Outer membrane ferric  siderophore receptor  (TonB-siderophore) | - |
| 3633 | 4000792..4001739 | 315 | ZP_16737333 from  *Pseudomonas syringae* pv.  *tabaci,*str. ATCC 11528*,*  5.00E-71 | pupR protein  (FecR) | - |
| 3634 | 4001736..4002245 | 169 | ZP_05636681 from  *Pseudomonas syringae* pv.  *tabaci,*str. ATCC 11528,  1.00E-35 | ECF subfamily RNA  polymerase sigma factor | - |
| 3635 | 4002430..4002549 | 39 | YP_005207902 from  *Pseudomonas fluorescens*  F113, 7.00E-11 | gamma-  glutamyltranspeptidase  (Ggt) | - |
|  |  |  |  |  |  |
| Cluster S12 (3753-3756) | | | | | |
| 3753 | 4148965..4150119 | 384 | YP_001022018 from  *Methylibium petroleiphilum*  PM1, 1.00E-155 | L-lactate dehydrogenase  (cytochrome) | - |
| 3754 | 4150212..4151129 | 305 | ZP_18357403 from  *Pseudomonas aeruginosa*  NCMG1179, 1.00E-31 | probable transcriptional  regulator | - |
| 3755 | 4151358..4152386 | 342 | YP_003017805 from  *Pectobacterium carotovorum*  subsp. *Carotovorum* PC1,  1.00E-113 | Spermidine/putrescine-  binding periplasmic  protein | - |
| 3756 | 4152742..4153779 | 345 | YP_006480604 from  *Pseudomonas aeruginosa*  DK2, 1.00E-134 | binding protein component of  ABC transporter  (PotD) | c55910 |
|  |  |  |  |  |  |
| Cluster S13 (3939-3945) | | | | | |
| 3939 | 4369492..4370796 | 434 | ZP_06941987 from  *Vibrio cholerae* RC385,  0.0 | McrBC 5-methylcytosine  restriction system  component | - |
| 3940 | 4370800..4371939 | 379 | ZP_16449700 from  *Escherichia coli* MS 16-3,  1.00E-174 | ATPase family protein | - |
| 3941 | 4371929..4372525 | 198 | VCRC385_02776 from  *Vibrio cholerae* RC385,  1.00E-102 | Transcriptional regulator | - |
| 3942 | 4372518..4374749 | 743 | ZP_06941990 from  *Vibrio cholerae* RC385,  0.0 | Type II restriction enzyme | - |
| 3943 | 4374746..4375840 | 364 | ZP_06941991 from  *Vibrio cholerae* RC385,  1.00E-173 | Adenine-specific DNA  methylase | - |
| 3944 | 4375837..4376118 | 93 | PACL_0211 from  *Pseudomonas aeruginosa,*  2.00E-21 | Predicted ATP-dependent  endonuclease of the OLD  family | - |
| 3945 | 4376733..4377776 | 347 | YP_960641 from  *Marinobacter aquaeolei*  VT8, 1.00E-127 | Restriction endonuclease | - |
|  |  |  |  |  |  |
| Cluster S14 (4407-4417) | | | | | |
| 4407 | 4928225..4930213 | 662 | ZP_17084331 from  *Pseudomonas fluorescens*  Q2-87, 0.0 | Nucleotide sugar  epimerase/dehydratase  WbpM | c43710 |
| 4408 | 4930267..4931283 | 338 | YP_261398 from  *Pseudomonas protegens* Pf-5,  1.00E-113 | Glycosyltransferase WbpL | c43720 |
| 4409 | 4931280..4932260 | 326 | YP_005093066 from  *Oceanimonas* sp. GK1,  1.00E-71 | NAD-dependent  epimerase/dehydratase  (WcaG) | c43730 |
| 4410 | 4932260..4933327 | 355 | PMI26_02321 from  *Pseudomonas* sp. GM33,  1.00E-116 | Glycosyltransferase | c43740 |
| 4411 | 4934187..4935092 | 301 | PMI26_02324 from  *Pseudomonas* sp. GM33,  1.00E-117 | Glycosyltransferases  involved in cell wall  biogenesis | c43750 |
| 4412 | 4935092..4936387 | 431 | PMI34_02001 from  *Pseudomonas* sp. GM74,  1.00E-127 | Membrane protein involved  in the export of O-antigen  and teichoic acid | c43760 |
| 4413 | 4936374..4937516 | 380 | YP_002261786 from  *Aliivibrio salmonicida*  LFI1238, 1.00E-143 | TDP-4-oxo-6-deoxy-D-  glucose transaminase  (WecE) | c43770 |
| 4414 | 4937976..4938200 | 74 | VISP3789_09613 from  *Vibrio splendidus* ATCC  33789, 0.25 | Chain length determinant  protein | - |
| 4415 | 4938574..4938909 | 111 | PMI31_05650 from  *Pseudomonas* sp. GM55,  7.00E-08 | Chain length determinant  protein | - |
| 4416 | 4939124..4939411 | 95 | ZP_05639882 from  *Pseudomonas syringae* pv.  *tabaci* str. ATCC 11528,  2.00E-48 | Integration host factor  subunit beta | c43780 |
| 4417 | 4939552..4939761 | 69 | YP_349789 from  *Pseudomonas fluorescens*  Pf0-1, 3.00E-24 | Lipoprotein | c43790 |

*The “-” refers to the absence of the homologue in CHA0.
