# Supplementary material for: Complete Genome Sequence of the Biocontrol Strain Pseudomonas protegens Cab57 Discovered in Japan Reveals Strain-Specific Diversity of This Species
Source: PLoS One. 2014 Apr 2;9(4):e93683. doi: 10.1371/journal.pone.0093683 (PMC3973561; doi:10.1371/journal.pone.0093683)
Supplement: Table S6 — Oligonucleotides used in this study. (DOCX) [file pone.0093683.s014.docx]

**Table S6.**

Oligonucleotides used in this study

| Oligonucleotide | Description | Source or reference |
| --- | --- | --- |
| RetSUF | 5'-TATGGATCCGGCCGAGGAAGGCAACGTCTA-3',  underlining indicates the artificial BamHI site | [15] |
| RetSUR | 5'-CGAAATCCCTTCGTTGGTTGA-3', anneals to the 5' region of RetSDF, located in the start codon region of *retS* | This study |
| RetSDF | 5’-TCAACCAACGAAGGGATTTCGCAGTTGAGCCGACAGGCTCTG -3', located in the stop codon region of *retS* | This study |
| RetSDR | 5'-TATAAAGCTTGACCCCGGTGAAGATGATCTG-3',  underlining indicates the artificial HindIII site | [15] |
| Phl2a | 5'-GAGGACGTCGAAGACCACCA-3' | [54] |
| Phl2b | 5'-ACCGCAGCATCGTGTATGAG-3' | [54] |
| PRND1 | 5'-GGGGCGGGCCGTGGTGATGGA-3' | [55] |
| PRND2 | 5'-YCCCGCSGCCTGYCTGGTCTG-3' | [55] |
| PltBf | 5'-CGGAGCATGGACCCCCAGC-3' | [56] |
| PltBr | 5'-GTGCCCGATATTGGTCTTGACCGAG-3' | [56] |
| PM2 | 5'-TGCGGCATGGGCGTGTGCCATTGCTGCCTGG-3' | [57] |
| PM7-26R | 5'-CCGCTCTTGATCTGCAATTGCAGGCC-3' | [57] |
